# CDK2 regulates the NRF1/*Ehmt1* axis during meiotic prophase I

**DOI:** 10.1083/jcb.201903125

**Published:** 2019-07-26

**Authors:** Nathan Palmer, S. Zakiah A. Talib, Chandrahas Koumar Ratnacaram, Diana Low, Xavier Bisteau, Joanna Hui Si Lee, Elisabeth Pfeiffenberger, Heike Wollmann, Joel Heng Loong Tan, Sheena Wee, Radoslaw Sobota, Jayantha Gunaratne, Daniel M. Messerschmidt, Ernesto Guccione, Philipp Kaldis

**Affiliations:** 1 Institute of Molecular and Cell Biology, Agency for Science, Technology and Research, Singapore; 2 Department of Biochemistry, National University of Singapore, Singapore

## Abstract

Meiosis generates four genetically distinct haploid gametes over the course of two reductional cell divisions. Meiotic divisions are characterized by the coordinated deposition and removal of various epigenetic marks. Here we propose that nuclear respiratory factor 1 (NRF1) regulates transcription of euchromatic histone methyltransferase 1 (EHMT1) to ensure normal patterns of H3K9 methylation during meiotic prophase I. We demonstrate that cyclin-dependent kinase (CDK2) can bind to the promoters of a number of genes in male germ cells including that of *Ehmt1* through interaction with the NRF1 transcription factor. Our data indicate that CDK2-mediated phosphorylation of NRF1 can occur at two distinct serine residues and negatively regulates NRF1 DNA binding activity in vitro. Furthermore, induced deletion of *Cdk2* in spermatocytes results in increased expression of many NRF1 target genes including *Ehmt1*. We hypothesize that the regulation of NRF1 transcriptional activity by CDK2 may allow the modulation of *Ehmt1* expression, therefore controlling the dynamic methylation of H3K9 during meiotic prophase.

## Abstract (precis)

Palmer et al. identify NRF1 as a novel CDK2 interactor and substrate. This interaction was found to be important for the DNA-binding activity of NRF1. Their findings demonstrate that the loss of CDK2 expression impairs the regulation of NRF1 transcriptional activity, leading to inappropriate transcription during meiotic division.

## Introduction

Prophase I is the longest and most complex stage of the first meiotic division, which can be further divided into five major sub-stages: leptotene, zygotene, pachytene, diplotene, and diakinesis (Cobb and Handel, 1998).

Progression through meiotic prophase I is driven in part by histone tail modifications, which direct specific proteins to interact with meiotic chromatin (Nottke et al., 2009; Kota and Feil, 2010). Chromatin modifications have been shown to be widespread and dynamic during germ cell development (Hammoud et al., 2014). Perhaps the best-known example of this is the designation of recombination hotspots during leptotene stage by the PR-domain zinc finger protein 9 (PRDM9). This enzyme is able to directly bind DNA through its C-terminal zinc fingers and catalyses the trimethylation of histone H3 at K4 and K36 (H3K4me3 and H3K36me3; Hayashi et al., 2005; Eram et al., 2014; Powers et al., 2016). This epigenetic signature is then associated with the formation of meiotic double strand breaks by the DNA topoisomerase SPO11 (Bergerat et al., 1997; Keeney et al., 1997; de Massy, 2013; Lange et al., 2016).

Another histone modification important for normal prophase I progression is the methylation of H3K9. The complex responsible for the establishment of dimethylated H3K9 is composed of the euchromatic histone methyltransferases (EHMT) EHMT1 and EHMT2 heterodimer (also known as GLP1 and G9a; Tachibana et al., 2005). During spermatogenesis, histone H3K9 dimethylation (H3K9me2) is established at specific sites in chromatin, as spermatogonia exit self-renewal and adopt a differentiating profile (Tachibana et al., 2007; Shirakawa et al., 2013). This persists throughout spermatogonial differentiation into primary spermatocytes and extends into the leptotene and zygotene sub-stages of prophase I, in which chromosomal homologues initiate pairing (also known as synapsis). During the pachytene stage, H3K9 becomes globally demethylated (H3K9me0; Tachibana et al., 2007), which occurs in tandem with the completion of chromosomal synapsis. The methylation status of H3K9 during this transitional period (especially in regard to di- and trimethylation) has been shown to be essential for normal synapsis of chromosomal homologues (Takada et al., 2011), but the upstream regulation of the epigenetic writers and erasers responsible for this transition is not known yet.

Here we provide compelling insights into the upstream regulatory process of chromatin regulation. We identify *Ehmt1*, a crucial regulator of H3K9me2 during the meiotic prophase, to be a target of a novel transcriptional regulatory pathway mediated by nuclear respiratory factor 1 (NRF1). Phosphorylation of NRF1 by CDK2 negatively regulates its binding to DNA. Induced deletion of CDK2 in male germ cells leads to enhanced binding of NRF1 to many promoters including *Ehmt1* and subsequently to inappropriately persisting levels of EHMT1 and its downstream histone mark (H3K9me2). We propose a regulatory role for CDK2 in negatively modulating NRF1 transcriptional activity during meiotic prophase. This allows NRF1 target genes such as *Ehmt1* to be turned off in a stage-specific manner during meiotic prophase I. Therefore, we propose that CDK2 regulates meiosis not only by tethering telomeres to the nuclear envelope (Viera et al., 2009, 2015; Mikolcevic et al., 2016; Tu et al., 2017) but also through the transcriptional regulation of NRF1.

## Results

### Regulation of H3K9me2 at the zygotene–pachytene transition

Since the completion of homologue synapsis occurs in near perfect coordination with the demethylation of H3K9 during pachytene stage of meiosis I (Tachibana et al., 2007), we set out to determine how this epigenetic switch might be affected in meiotic arrest models with synapsis defects. For this purpose, we chose *Sun1KO* (Ding et al., 2007), *Prdm9KO* (Hayashi et al., 2005), *Speedy AKO* (Mikolcevic et al., 2016; Tu et al., 2017), *Cdk2KO* (Viera et al., 2009, 2015), *Cdk2^D145N^* knockin (kinase-dead mutant; Chauhan et al., 2016), and *Cdk2^T160A^* knockin (nonactivatable mutant that can form active complexes with Speedy A but not with cyclins; Cheng et al., 2005; Chauhan et al., 2016) mice for closer analysis.

**Figure 1.**
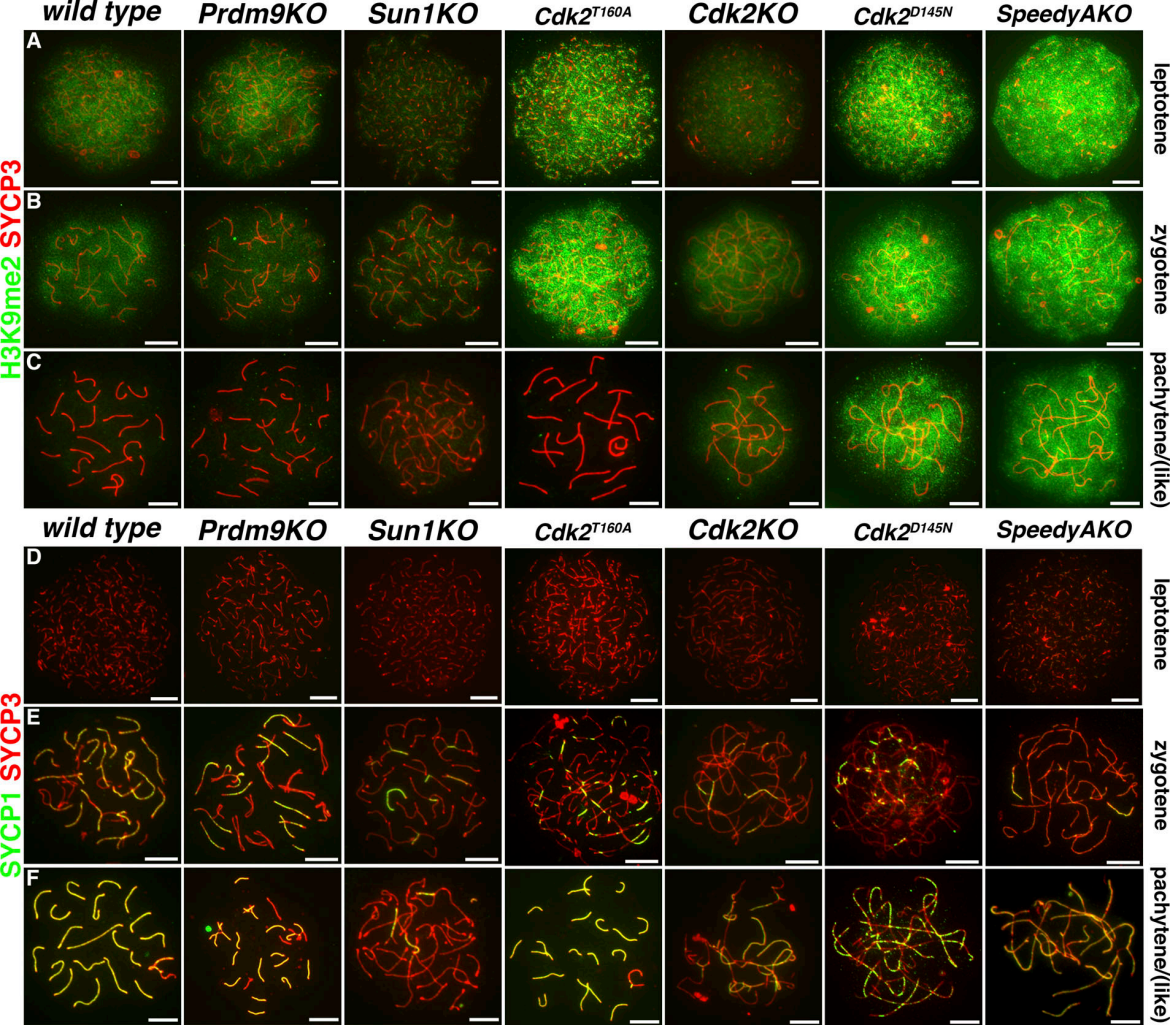
**H3K9me2 expression is abnormally retained in pachytene stage mutant spermatocytes.** Immunostaining of P18 meiotic chromosome spreads from wild-type, *Prdm9KO*, *Sun1KO*, *Cdk2^T160A^*, *Cdk2KO*, *Cdk2^D145N^*, *and Speedy AKO* mice. Representative images of leptotene, zygotene, and pachytene stages (or the closest possible stage) of prophase I of the first meiotic division according to wild type are shown as indicated (top to bottom). For *Prdm9KO*, *Sun1KO*, *SpeedyAKO*, *Cdk2KO*, and *Cdk2^D145N^*, meiotic arrest of spermatocytes occurs before the completion of pachytene, whereas *Cdk2^T160A^* show normal synaptonemal complex dynamics during early meiotic prophase I, but arrest later due to an unrelated meiotic defect (unpublished data). In A–C, costaining of SYCP3 (red) and H3K9me2 (green) is shown, whereas in D–F, costaining of SYCP3 (red) and SYCP1 (green) is shown. At least 50 images of distinct spermatocytes were analyzed for each genotype and stage for each costaining. Original images were individually pseudo-colored and combined as indicated using Adobe Photoshop CC 2018. Scale bars, 5 µm.

We prepared meiotic chromosome spreads from P18 mouse testis during the synchronous first wave of spermatogenesis to determine H3K9me2 levels and distribution. Spreads were immunostained for H3K9me2 and SYCP3 (Fig. 1, A–C) or SYCP1 and SYCP3 (Fig. 1, D–F). SYCP3 was used to monitor progression through the sub-stages of prophase I via the extent of its localization to synapsing chromosomes. During the leptotene and zygotene stages of prophase I, H3K9me2 levels were indistinguishable between both wild type and each of the mutant mouse models that we used. Here the H3K9me2 signal could be observed as a cloud-like staining, indicating broad coverage of this histone mark on chromatin (Fig. 1, A and B). This suggested that the establishment of high levels of H3K9me2 in early spermatocytes occurs as previously described (Tachibana et al., 2007). In wild-type, *Prdm9KO*, *Sun1KO*, and *Cdk2^T160A^* spermatocytes, the H3K9me2 signal was undetectable at pachytene or—for the meiotic arrest models—pachytene-like stage (Fig. 1 C; Tachibana et al., 2007), suggesting the loss of H3K9 dimethylation specifically at this stage. Remarkably, however, in *SpeedyAKO, Cdk2KO*, and *Cdk2^D145N^* mutants, high levels of H3K9me2 persisted in pachytene-like spermatocytes (Fig. 1 C).

The failure of these mutant spermatocytes to reduce H3K9me2 levels during meiotic prophase suggests that Speedy A, CDK2, and the catalytic activity of CDK2 are important for this transition. The requirement for catalytic activity of CDK2 was also suggestive that phosphorylation of a CDK2 substrate might be a potential effector driving this transition. Additionally, the observation of an unaffected H3K9me2 high-to-low transition in *Cdk2^T160A^* spermatocytes further suggests that CDK2/cyclin (as a difference to CDK2/Speedy A) complex formation is not required for this process and is consistent with the normal patterns of synapsis observed in pachytene stage *Cdk2^T160A^* spermatocytes.

### Localization of EHMT1 and H3K9me2 in testis

**Figure 2.**
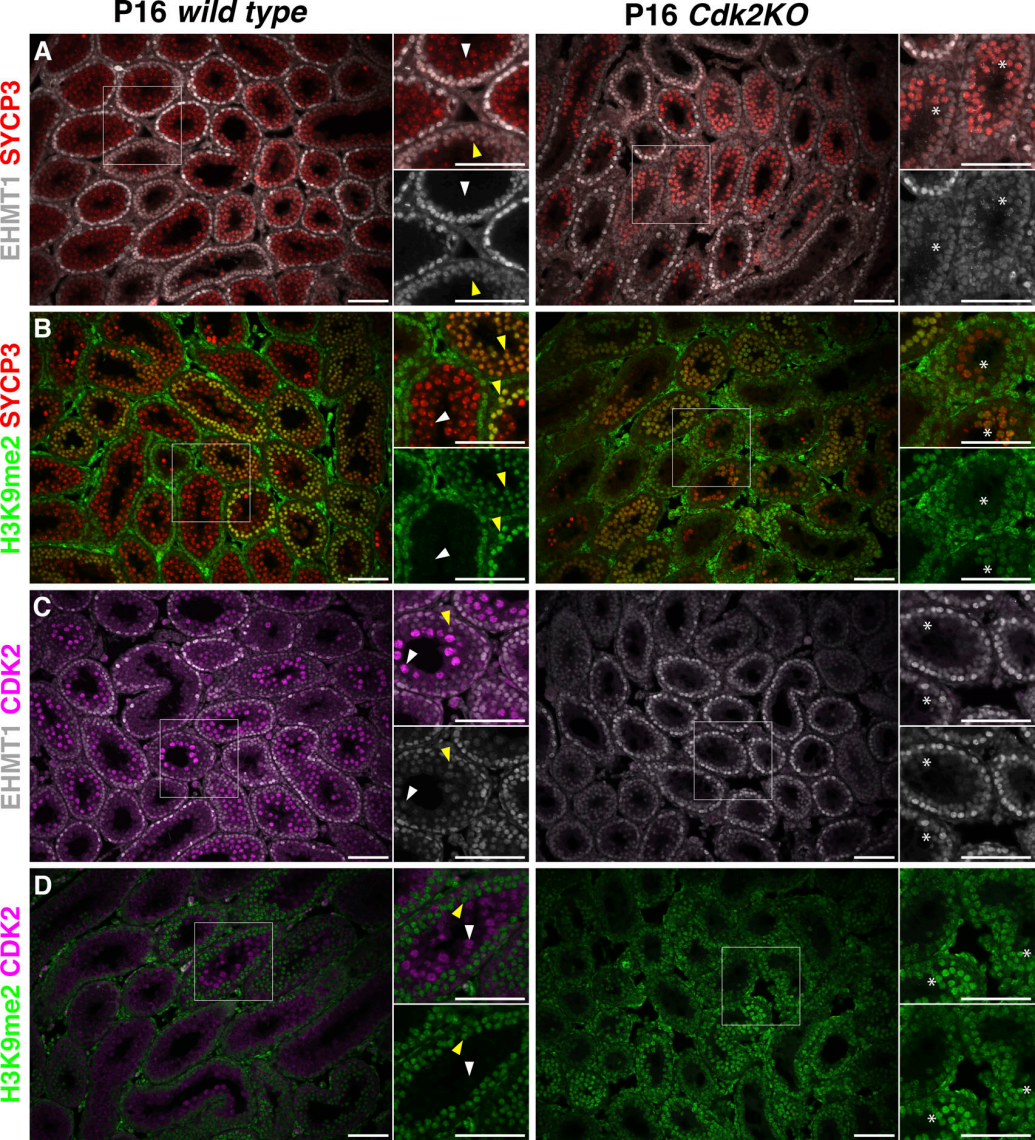
**Immunofluorescence analysis of P16 wild-type and *Cdk2KO* testis.** Immunostaining was performed on P16 testis sections from wild-type (left) and *Cdk2KO* mice (right). Double immunostaining of EHMT1 (white) or H3K9me2 (green) with either SYCP3, a marker of spermatocytes for all stages of meiotic prophase I (red; A and B), or CDK2 (purple; C and D). For each image, areas of interest are highlighted (white boxes) and displayed as magnified images toward the upper right-hand side. Additional single channel images for either EHMT1 or H3K9me2 are also shown to the lower right-hand side. This is to indicate where costaining has occurred in the merged image. For wild-type images, yellow arrowheads indicate early prophase I (preleptotene, leptotene, and zygotene) spermatocytes, and white arrowheads indicate pachytene stage spermatocytes. For *Cdk2KO* images, potential pachytene-arrest stage spermatocytes are indicated by white asterisks. At least 25 images of distinct areas of seminiferous tubules were analyzed for each genotype and for each costaining. Original images were individually pseudo-colored and combined as indicated using Adobe Photoshop CC 2018. Scale bars, 50 µm.

To confirm the mis-regulation of H3K9me2 and EHMT1 expression, we used an immunostaining approach to identify specific cell types contributing to the increased levels of H3K9me2 observed in *Cdk2KO* testis. Immunostaining of testis sections from P16 wild-type and *Cdk2KO* mice using antibodies against EHMT1 or H3K9me2 in combination with either SYCP3 (a marker of spermatocytes of all stages of meiotic prophase I) or CDK2 was performed (Fig. 2). At postnatal day 16 (P16), spermatogenic cells of both wild-type and *Cdk2KO* animals are synchronised within the first wave of spermatogenesis (Bellvé et al., 1977) and are therefore more comparable than adult mice in terms of cellularity due to the lack of widespread spermatocyte cell death that occurs in adult *Cdk2KO* testes. In wild-type testis, EHMT1 was observed in somatic cells, spermatogonia, and early prophase I spermatocytes (yellow arrows, inset of Fig. 2 A), but this signal was completely lost by the pachytene stage of prophase I (white arrows, inset of Fig. 2 A; Tachibana et al., 2007; Di Giacomo et al., 2013; Zamudio et al., 2015). In *Cdk2KO* testis, however, we found EHMT1 to be largely colocalized with SYCP3 in spermatocytes (Fig. 2 A). This indicates an expansion of EHMT1 protein expression into a pachytene arrest stage during meiotic prophase I (asterisks, inset of Fig. 2 A). The localization of H3K9me2 in wild-type testis mirrored that of EHMT1 as previously reported (Tachibana et al., 2007), and an expansion of expression could be observed in *Cdk2KO* mice (Fig. 2, B and D). Interestingly, costaining of EHMT1 or H3K9me2 with CDK2 revealed a mutually exclusive expression pattern whereby upon CDK2 expression, the levels of EHMT1 and H3K9me2 are reduced (Fig. 2, C and D). These results indicate a possible regulatory relationship between CDK2, EHMT1, and H3K9me2.

### Increased levels of Ehmt1 at both the mRNA and protein level in testis

**Figure 3.**
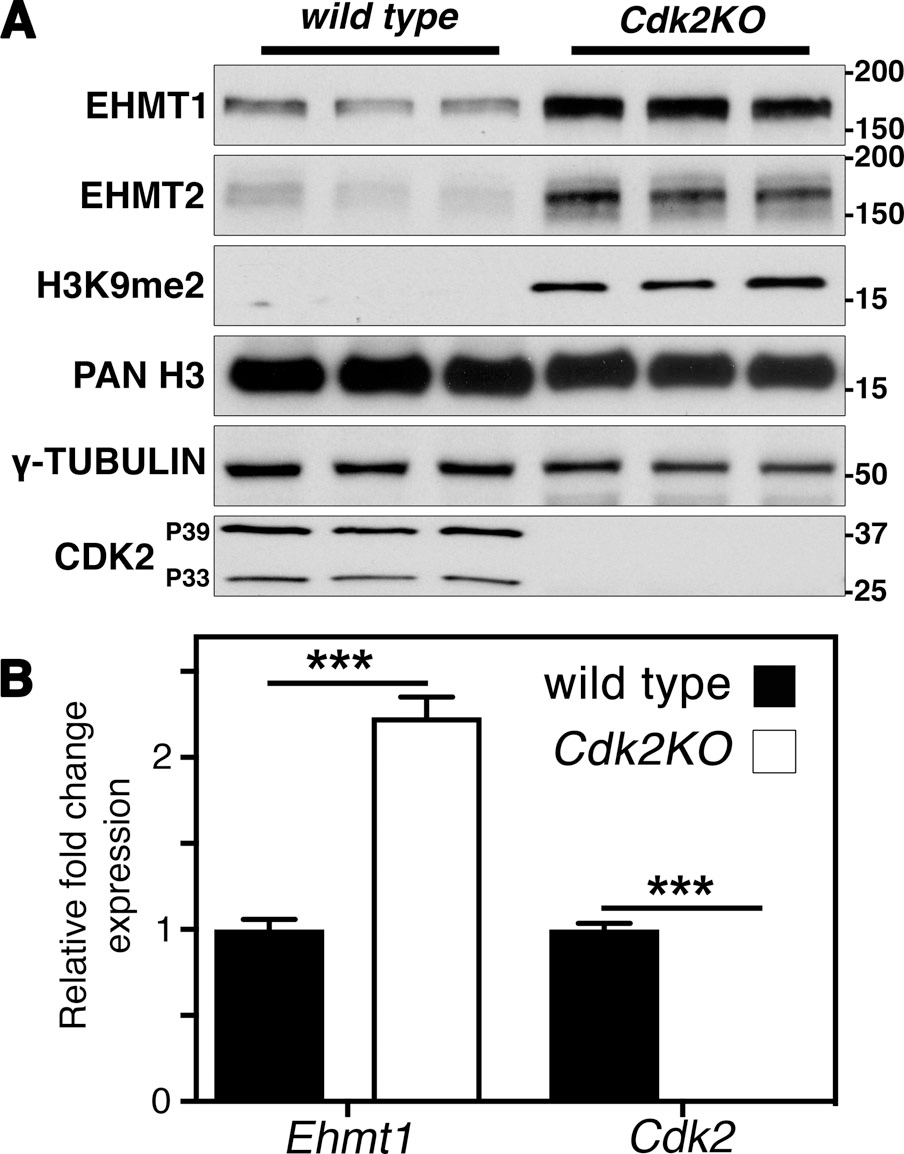
**EHMT1 expression and H3K9me2 levels are increased in *Cdk2KO* testis. (A)** Western blotting of EHMT1, EHMT2, H3K9me2, pan-histone H3, γ-tubulin, and CDK2 in P56 wild-type and *Cdk2KO* testis (three biological replicates are shown for each). The expression of EHMT1 and EMHT2 at the protein level was increased in *Cdk2KO* testis compared with wild-type. This was also associated with an increase in H3K9me2 levels. Pan-histone H3 is displayed as a loading control. **(B)** qPCR analysis of P56 wild-type and *Cdk2KO* testis. Expression is displayed as fold change normalized to the expression of the eEF2 housekeeping gene. Error bars are representative of the SD of normalized fold change values from at least three biological replicates as compared to wild type. Gene expression between biological replicates was assumed to follow a normal distribution but this was not formally tested. The levels of *Ehmt1* transcript are significantly increased as compared with wild-type (***, P < 0.001, as determined by unpaired *t* test).

We found an increase of H3K9me2 in pachytene and an expanded localization of EHMT1 in *Cdk2KO* testis (Fig. 1 and Fig. 2). To address whether EHMT1 is regulated on the level of activity, protein stability, or mRNA levels, we performed Western blots and RT–quantitative PCR (qPCR) using wild-type and *Cdk2KO* testis. Western blot analysis indeed revealed increased EHMT1 protein levels in *Cdk2KO* testis resulting in globally increased dimethylation of histone H3 on lysine 9 (Fig. 3 A). This is consistent with our immunofluorescence results (Fig. 1 and Fig. 2). As levels of *Ehmt1* transcript were also increased by 2.6-fold in *Cdk2KO* testis as compared with wild-type (Fig. 3 B), we conclude that *Ehmt1* is most likely regulated, directly or indirectly, by CDK2 at the transcriptional level.

The mechanisms repressing EHMT1 and EHMT2 expression specifically during the zygotene–pachytene transition are unknown (Tachibana et al., 2007; Shirakawa et al., 2013), yet they are thought to be crucial for the transition to a H3K9me2-low state. Since the transcription factor promoting the expression of *Ehmt1* is not known and since we had a model system in which *Ehmt1* transcription is deregulated (*Cdk2KO*), we hypothesized that CDK2 might influence the expression of *Ehmt1* by regulating its transcription through unknown mechanisms.

### CDK2 is chromatin-bound to NRF1 sites during meiosis

CDK2 lacks a DNA-binding domain and is not known to interact with chromatin directly. Instead, CDK2 regulates transcription during the mitotic cell cycle through the phosphorylation of E2F (Xu et al., 1994; Kitagawa et al., 1995; Cheng et al., 2000; Morris et al., 2000) and its repressor, the retinoblastoma protein (Lundberg and Weinberg, 1998; Dyson, 2016). We hypothesized that a similar pathway might control *Ehmt1* during meiosis.

**Figure 4.**
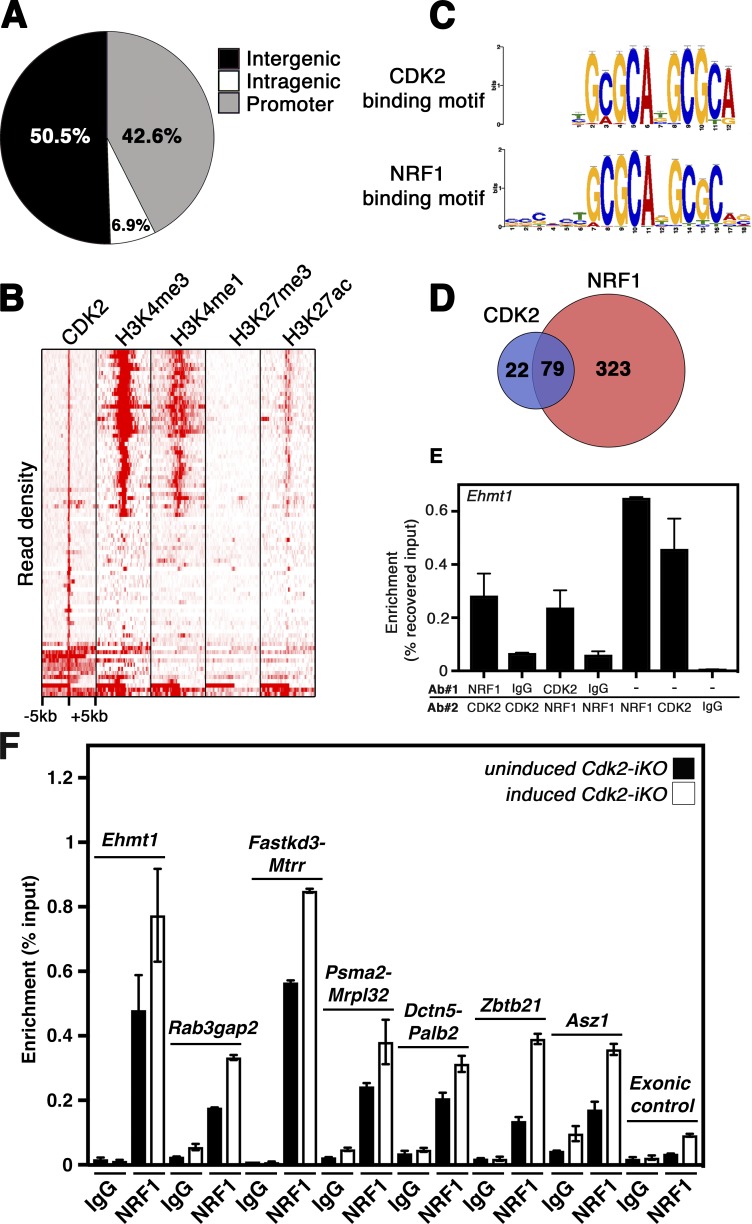
**CDK2 binds to chromatin at promoter regions enriched for NRF1. (A)** Pie chart indicating the percentages of CDK2 bound genomic loci found within intergenic, intragenic, or promoter regions. Promoter regions here are defined as within ±5 kb of a transcription start site. **(B)** Density plot of mapped reads centered on genomic loci positive for CDK2 binding. Density plots of histone mark occupation from isolated spermatocyte DNA are shown for comparison (Hammoud et al., 2014). The majority of CDK2-bound regions are occupied by the epigenetic marks of active transcription (H3K4me3, H3K4me1, and H3K27ac) but not by the transcriptionally repressive H3K27me3 mark. Scale shows ±5 kb relative to the center of CDK2-bound sites. **(C)** Motif analysis of genomic loci positive for CDK2 binding: The over-represented motif from CDK2-bound loci as generated by MEME suite (top) and NRF1-binding motif extracted from JASPAR database of transcription factor binding motifs (bottom). The significance of the identified CDK2 binding motif to the NRF1 binding motif as determined by GREAT INPUT (McLean et al., 2010) is P = 6.7444 × 10^−6^. **(D)** Venn diagram illustrating the overlap of CDK2-bound and NRF1-bound genomic regions from CDK2 and NRF1 ChIPseq analysis in testis. **(E)** Representative NRF1/CDK2 ChIP-reChIP analysis of the *Ehmt1* promoter via RT-qPCR. Two ChIP-reChIPs were performed using either CDK2 or NRF1 antibodies followed by the reciprocal antibody as indicated. Control immunoprecipitations were also performed using nonspecific rabbit IgG followed by CDK2 or NRF1 antibodies. Immunoprecipitated genomic DNA was used for RT-qPCR directed against the region of the *Ehmt1* promoter identified as bound by both CDK2 and NRF1 by ChIPseq. **(F)** Representative ChIP-qPCR for multiple genomic loci using antibodies against NRF1 or nonspecific rabbit IgG for immunoprecipitation. Enrichment of specific NRF1 binding to genomic loci is shown relative to input. Each ChIP was performed on chromatin extracted from corn oil–treated (black bars) and tamoxifen-treated (white bars) *Cdk2-iKO* testis at P68, 12 d after corn oil/tamoxifen treatment (started at P56). CDK2 and NRF1 ChIPseq experiments were performed using pooled spermatogenic cells from distinct groups of animals (biological replicates) at least three times with similar results. One representative dataset was analyzed to create these figures, as shown in Tables S1 and S3, respectively. ChIP-reChIP and ChIP-qPCR experiments were performed using pooled spermatogenic cells from distinct groups of animals (biological replicates). These experiments were repeated at least four times for each genomic loci analyzed, with similar results. Error bars for ChIP-reChIP data are representative of the SD of at least three technical replicates of the same experiment.

Although the functions of CDK2 as a transcriptional regulator during meiotic division is yet to be investigated, we hypothesized that such a role might allow CDK2 to indirectly modulate the levels of *Ehmt1* transcription during meiotic prophase. To test for potential sites of CDK2 interaction with chromatin in testis, we used an unbiased genome-wide approach. Chromatin immunoprecipitation (ChIP) and sequencing (ChIPseq) using antibodies against CDK2 was performed on primary cells extracted from wild-type testis. We identified 101 high-confidence (fold change greater than three) CDK2-bound genomic regions of which >40% (43/101) were localized at promoter regions within 5 kb of transcription start sites (Fig. 4 A and Table S1). This indicated indeed that CDK2 could bind to chromatin. A number of the CDK2-bound genes identified have already been shown to be important for fertility, and several knockout mouse models of these genes result in meiotic arrest or defects occurring within meiotic prophase I. These include *Rnf8* (Li et al., 2010), *Msh4* (Kneitz et al., 2000), *Dazap1* (Hsu et al., 2008), *Spata22* (La Salle et al., 2012), *Asz1* (Ma et al., 2009), *Smarca4* (Kim et al., 2012; Wang et al., 2012), and *Ndrg3* (Pan et al., 2017a). Several CDK2-bound genes have also been implicated in other aspects of germ cell development, including *Palb2* (Simhadri et al., 2014; Hartford et al., 2016), *Pds5b* (Zhang et al., 2007), *Safb1* (Ivanova et al., 2005), and *Herc4* (Rodriguez and Stewart, 2007), or have been investigated as potential markers of infertility in human populations: *Mtrr* (Lee et al., 2006; Liu et al., 2015a) and *Vdac3* (Pan et al., 2017b).

Overlaying CDK2-bound regions with H3K4me1/3 and H3K27ac (active marks) or H3K27me3 (repressive mark) histone modifications in spermatocytes (Hammoud et al., 2014) suggested that promoter-bound CDK2 was almost always associated with euchromatic promoters (Fig. 4 B). Surprisingly, however, CDK2-bound regions did not show substantial overlap with E2F transcription factor binding sites identified previously in mouse embryonic fibroblasts (deposited in GEO under accession no. GSM1833208; Kent et al., 2016; Fig. S1, A and B; and Table S2). In accordance with this finding, we observed that CDK2-bound regions were associated with a significantly enriched binding motif, different from previously identified E2F binding motifs (Tao et al., 1997). This motif matched the experimentally determined predicted motif of the NRF1 transcription factor (Fig. 4 C). NRF1 is a ubiquitously expressed transcription factor primarily associated with the expression of genes essential for mitochondrial respiration (Kelly and Scarpulla, 2004; Jornayvaz and Shulman, 2010). Interestingly, this essential transcription factor is detected at high levels in testis (Escrivá et al., 1999). While we were completing our study, a testis-specific *Nrf1* knockout in mice was shown to cause apoptotic loss of germ cells (Wang et al., 2017). Since the loss of *Nrf1* was associated with defects in spermatogonial stem cell proliferation and differentiation, the role of NRF1 during the subsequent meiotic divisions requires further investigation.

Enrichment of the NRF1 binding motif at CDK2-bound genomic regions prompted us to address whether these sites are indeed shared with NRF1. We therefore performed NRF1 ChIPseq in wild-type mouse testis under the same experimental conditions as CDK2 and identified 481 high-confidence NRF1-bound genomic loci (Table S3). The specificity of the pulldown was confirmed by the pronounced representation of NRF1 binding motifs within bound regions. 83% of these loci were associated with promoter regions (402/481) and displayed a high degree of overlap with NRF1 target genes identified in a recently reported NRF1 ChIPseq on whole testis lysate (Wang et al., 2017; Fig. S1 C and Table S4). Most strikingly, however, nearly 80% of the identified CDK2-bound loci (79/101) and 93% of the promoter-associated (40/43) CDK2-bound regions were also identified as bound by NRF1 (Fig. 4 D and Table S5), suggesting that NRF1 and CDK2 bind to the same loci. To determine whether NRF1 was binding to promoter regions of genes within common pathways, we performed an ontological analysis, which indicated that genes bound by NRF1 were mainly involved in meiosis (Fig. S1 D, marked by red bars; and Table S6). Interestingly, NRF1-bound genomic loci were also significantly associated with a mouse phenotype of arrest of spermatogenesis (Table S6), further suggesting that NRF1 may regulate genes important for fertility. Surprisingly, however, biological processes typically associated with NRF1 in other tissues, such as metabolism or mitochondrial respiration (Kelly and Scarpulla, 2004; Jornayvaz and Shulman, 2010), were not significantly enriched, supporting a compelling argument for testis-specific NRF1 functions in regulating meiosis directly.

To distinguish whether CDK2 and NRF1 were binding to chromatin independently or together, we performed sequential ChIP (ChIP-reChIP) using NRF1 and CDK2 antibodies on adult wild-type testis. We observed that NRF1 immunoprecipitation followed by CDK2 immunoprecipitation (and vice versa) enriched for genomic loci identified as cobound by both CDK2 and NRF1, confirming simultaneous binding of these two proteins to the same loci (one example: the promoter of the *Ehmt1* gene is shown in Fig. 4 E). Enrichment was also observed at additional identified loci (Fig. S2, A and B), and no enrichment was seen for an exonic negative control region (Fig. S2 C), confirming that CDK2 and NRF1 bind to the same loci.

To validate our results, we performed NRF1 ChIP-qPCR in an inducible germ cell–specific *Cdk2* knockout model (*Cdk2-iKO*; see Fig. S3 and Materials and methods for a detailed description of this model) to determine the extent of NRF1 binding to target promoters identified in our earlier ChIPseq experiments. We chose five CDK2/NRF1-bound regions, two regions that were only bound by NRF1 (according to ChIPseq), as well as an unbound locus as a negative control (Fig. 4 F). In the uninduced *Cdk2-iKO* (corn oil–treated control), NRF1 bound to all these regions with the exception of the negative control. 12 d after tamoxifen injection, NRF1 binding was enhanced at all seven tested regions in the induced *Cdk2-iKO*, suggesting that CDK2 might bind to all NRF1 target regions. Given that CDK2 and NRF1 bind to chromatin at the same time (Fig. 4 E), our data suggest a negative regulation of NRF1 binding by CDK2 in the wild type. The enrichment of NRF1 binding to target promoters in the absence of *Cdk2* was reproducible between biological replicates and ranged from an average of 1.3–2.3-fold higher levels of binding at each locus (data not shown).

### Physical NRF1/CDK2 interactions

**Figure 5.**
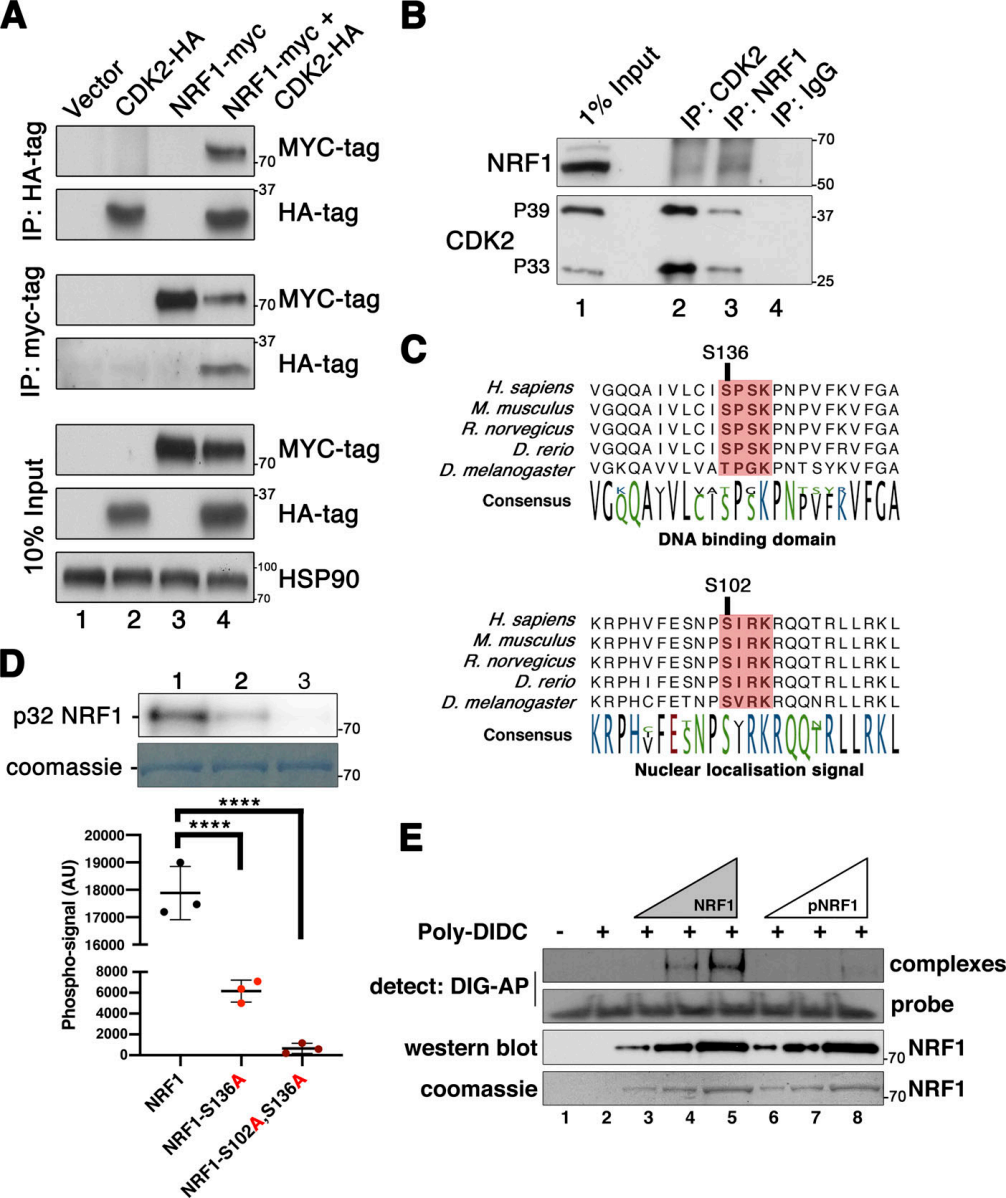
**NRF1 binds to CDK2 and is a novel CDK2 substrate in vitro. (A)** HEK-293T cells expressing either empty vector control, NRF1-MYC, or CDK2-HA, or coexpressing NRF1-MYC and CDK2-HA were lysed as described. NRF1-MYC could be coimmunoprecipitated with CDK2-HA and vice versa (lane 4). Bottom panels show Western blots of 10% of total protein lysate used for immunoprecipitation (Input). HSP90 was used as a loading control. **(B)** Western blotting of immunoprecipitates from whole testis lysate. Lane 1: 1% of input (16 µg of whole testis lysate); lane 2: IP CDK2; lane 3: IP NRF1; lane 4: IP nonspecific IgG. Coimmunoprecipitation can be seen in lanes 2 and 3 of both of the upper and lower panels. **(C)** Evolutionary conservation of the amino acid sequence surrounding serines 102 and 136 of NRF1 in multiple species. Potential CDK phosphorylation sites are highlighted in pink. **(D)** Representative kinase assay using 1 µg of wild-type (lane 1), S136A mutant (lane 2), or S102A/S136A (lane 3) full-length NRF1–GST fusion proteins as a substrate of CDK2/cyclin A in the presence of radiolabeled ATP (upper panel). Quantification of the corresponding phospho-signal was done by PhosphoImager using the Multi Gauge software (Ver3.X) from three replicate experiments as displayed below. Significance was determined by one-way ANOVA. Data were assumed to be normally distributed, but this was not formally tested (****, P < 0.0001). Error bars are representative of the SD of phospho-signal values from at least three technical replicates of the same experiment. Equal protein loading is shown by Coomassie staining of the dried gel (lower panel). **(E)** EMSA using a probe of the *Ehmt1* promoter. A DIG-labeled *Ehmt1* promoter probe was incubated with increasing concentrations of either CDK2/cyclin A2 phosphorylated NRF1 (pNRF1; lanes 6–8; 1, 2, or 3 µg, respectively) or nonphosphorylated NRF1 (lanes 3–5; 1, 2, or 3 µg, respectively). “Probe only” and “Probe + Poly DIDC only” conditions are shown in lanes 1 and 2, respectively. Shifted probe (complexes) or free probe (probe) were detected via anti-DIG-alkaline phosphatase (AP) antibody treatment followed by chemiluminescent detection of alkaline phosphatase. Protein loading is shown by both Western blotting and Coomassie staining of NRF1 and pNRF1 on separate SDS-PAGE gels. All experiments were repeated at least three times with similar results. One representative image is shown in each figure. AU, arbitrary units.

We next explored the potential physical interactions between CDK2 and NRF1 using a different experimental approach than ChIP-reChIP. To this end, we coexpressed tagged MYC-NRF1 with HA-CDK2 in human cell lines. Upon immunoprecipitation of MYC-NRF1, we found prevalent binding of HA-CDK2 (and vice versa), demonstrating the interaction of these proteins (Fig. 5 A).

To further investigate the biological relevance of this interaction, we performed immunoprecipitation of endogenous CDK2 or NRF1 directly from wild-type mouse testis lysates (Fig. 5 B). We were able to detect the coimmunoprecipitation of NRF1 with CDK2, confirming the existence of a complex between these proteins. In testis, CDK2 can be detected as two distinct isoforms, which appear at a molecular weight of ≈33 and 39 kD, respectively, which arise due to alternative splicing of exon 6 (Ellenrieder et al., 2001). Interestingly, NRF1 was able to coimmunoprecipitate with both of these isoforms. Our previous studies have indicated that 39-kD CDK2 (p39) is expressed preferentially in pachytene-stage spermatocytes (Tu et al., 2017), suggesting that at least some of the detected interaction may occur in this cell type.

The functions of CDK2 during meiotic prophase are linked to its kinase activity since mice expressing catalytically inactive *Cdk2^D145N^* mutant protein in a homozygous manner are infertile (Chauhan et al., 2016). To test whether NRF1 might be a direct substrate of CDK2, recombinant GST-NRF1 was incubated with activated CDK2/cyclin A2 kinase complexes in the presence of ATP. The resulting putative phospho-protein was analyzed by phospho-epitope mapping using phosphoserine/threonine enrichment following mass spectrometry analysis by two independent laboratories. We identified two residues in NRF1 (serines 102 and 136) that were phosphorylated by CDK2 (MS2 spectra of one replicate for both identified phosphopeptides is shown in Fig. S4, A and B). The predicted CDK phosphorylation site and the conservation of this motif in multiple species are shown in Fig. 5 C.

To validate these phosphorylation sites as CDK2 substrates in vitro, we produced recombinant full-length NRF1 proteins carrying serine to alanine mutations at S136 or S136/S102 in combination. Kinase assays were then performed to test their phosphorylation levels following incubation with active CDK2/cyclin A2 kinase complexes in the presence of radiolabeled ATP (γ^32^P-ATP). While we observed that wild-type NRF1 protein could efficiently incorporate radiolabeled ATP, point mutation of S136 and S136/S102 in combination reduced the observed phospho-signal by 63% and 93%, respectively (Fig. 5 D). These data were supported by additional CDK2 kinase assays directed against short GST-fusion proteins containing only the CDK consensus sites for S102 and S136, which were both positive for CDK2 phosphorylation. In contrast, we found no CDK2 activity toward similar proteins containing S47 (Fig. S4, C and D), a site that was previously reported to be phosphorylated by cyclin D–containing CDK complexes (Wang et al., 2006).

Interestingly, S136 is an evolutionarily conserved CDK phosphorylation site (SPSK; Holmes and Solomon, 1996) located within the DNA-binding domain of NRF1. In contrast, S102 is a noncanonical motif (SIRK), yet it is conserved across species and found within the nuclear localization signal of NRF1 (Fig. 5 C), suggesting that these two phosphorylation sites may serve different functional purposes.

### NRF1 binding to target loci is increased in testis lacking CDK2

Our finding of a CDK2-directed phospho-site within the DNA binding domain of NRF1 (serine 136) was suggestive that binding and phosphorylation of NRF1 by CDK2 could result in altered NRF1 transcriptional activity. To investigate the direct binding of recombinant NRF1 to DNA, we performed electrophoretic mobility shift assays (EMSAs) using a digoxigenin (DIG)-labeled 45mer probe corresponding to putative NRF1 binding sites within a target promoter. For simplicity, we used the *Ehmt1* promoter as one of the NRF1 targets. The regions bound by NRF1 and CDK2 identified in our ChIPseq studies (Table S5 and Fig. S5) were included in the probe used for the EMSA assay. Recombinant NRF1 protein (purified from bacteria and therefore unphosphorylated) bound to the probe in a concentration-dependent manner (Fig. 5 E) as expected. When NRF1 was prephosphorylated by CDK2/cyclin A2 (leading to phosphorylation of S102 and S136 as we have shown previously), it bound less prominently to the probe compared with nonphosphorylated NRF1 (Fig. 5 E, compare lanes 3–5 to 6–8). This indicates that the binding of NRF1 to target DNA in vitro is less efficient after phosphorylation by CDK2, which could result in changes of NRF1 target expression.

### NRF1 regulates the mRNA expression of many genes in spermatocytes

**Figure 6.**
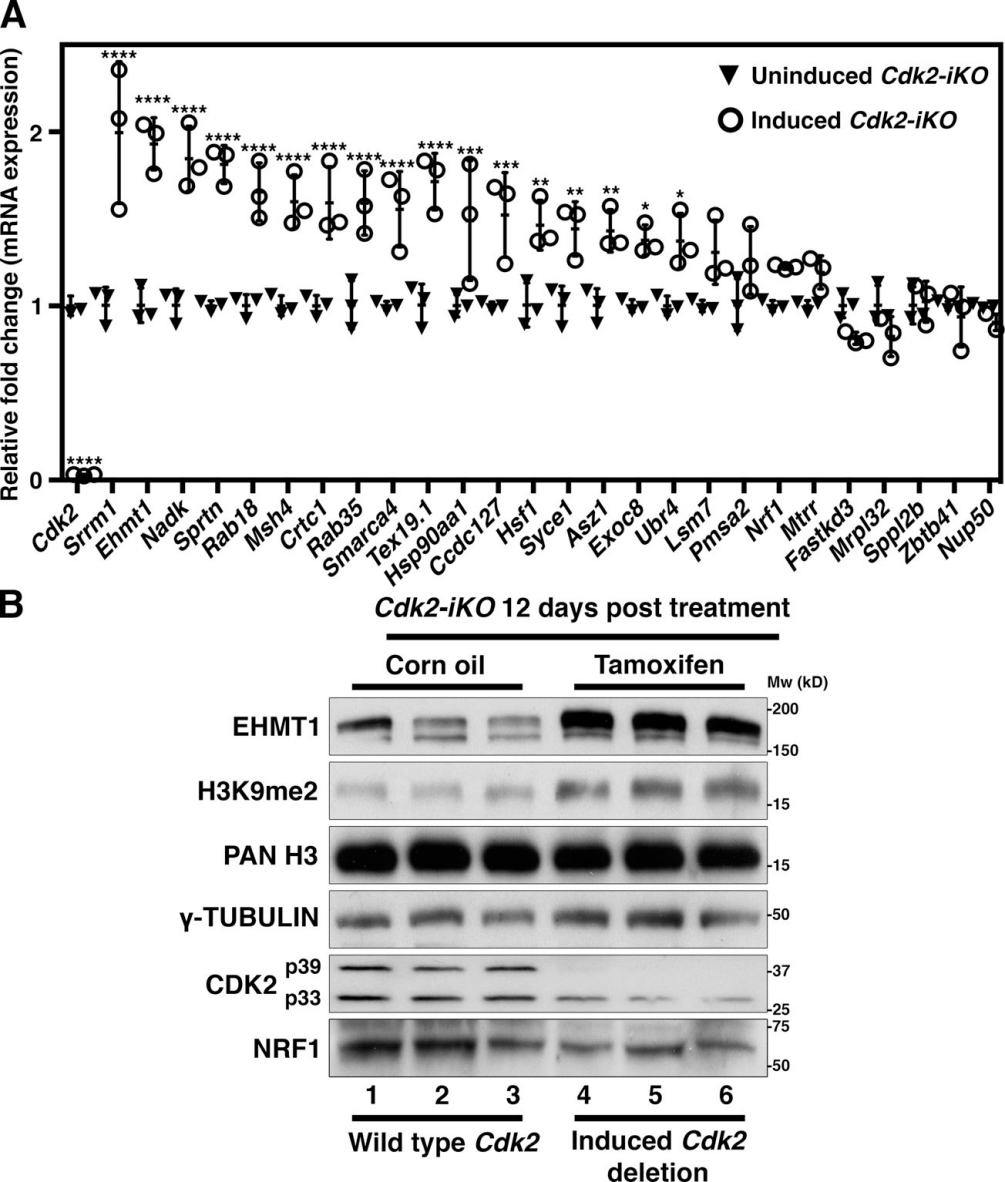
**NRF1 target mRNA expression is increased upon *Cdk2* deletion. (A)** qPCR analysis of spermatocyte cDNA isolated from corn oil treated (black triangles) and tamoxifen treated (white circles) *Cdk2-iKO* mice—referred to as noninduced and induced *Cdk2-iKO*, respectively. Expression is displayed as fold change normalized to the expression of the *eEF2* housekeeping gene. STA-PUT spermatocyte isolation (as described in Materials and methods) for this experiment was performed at P68, 12 d after corn oil/tamoxifen treatment (started at P56). 17/25 NRF1 target genes showed significant increases in expression. Three biological replicates were tested for each group for each gene. Gene expression of each biological replicate was assumed to be normally distributed, but this was not formally tested. Error bars are representative of the SD of normalized fold change values from at least three biological replicates as compared to noninduced *Cdk2-iKO* controls. Significance was determined via unpaired *t* test (****, P ≤ 0.0001; ***, P < 0.001; **, P < 0.005; *, P < 0.05). **(B)** Western blotting of NRF1, EHMT1, and H3K9me2 in corn oil–treated *Cdk2-iKO* (lanes 1–3) or tamoxifen-treated *Cdk2-iKO* (lanes 4–6) whole testis lysates. For *Cdk2-iKO* animals, testis isolation for this experiment was performed at P68, 12 d after corn oil/tamoxifen treatment (started at P56). γ-Tubulin and pan-histone H3 (PAN H3) are shown as loading controls.

To further test how *Cdk2* loss affects the expression of NRF1 targets, we determined the mRNA expression of a wide range of genes identified as targets from our NRF1 ChIPseq experiments. From a total of 402 NRF1-bound gene promoters, 25 genes were selected, some of which had prior known functions in meiosis. *Nrf1* and *Cdk2* were also included as controls. For this experiment, spermatocytes were isolated from whole testis using the STA-PUT methodology, which uses a linear gradient of BSA to separate spermatogenic cells based on their size and mass (see Materials and methods). After isolation, mRNA expression was measured 9 or 12 d after tamoxifen injection, at a time when CDK2 mRNA and protein levels were experimentally determined to be depleted (see Fig. S7 A). Importantly, at this time point the histology of testis sections was normal, and few apoptotic cells were detected (quantified in Fig. S3 A). At 9 d after tamoxifen injection, most genes tested displayed no significant change in expression when compared with control mice with the exception of *Ehmt1*, *Tex19.1*, *Msh4*, and, not surprisingly, *Cdk2* itself (Fig. S7 A). In contrast, by 12 d after tamoxifen injection, 17 out of 25 genes displayed increased mRNA expression after deletion of *Cdk2* (Fig. 6 A), indicating that CDK2 indirectly affects the expression of NRF1 target genes. In addition, when gene expression was monitored after corn oil injection, it was slightly decreased at 12 d compared with 9 d, indicating that the increase in gene expression after deletion of *Cdk2* was not due to a global increase between 9 and 12 d (data not shown). Importantly, to exclude the possibility of tamoxifen treatment disrupting spermatogenesis and thus resulting in changes to gene expression, we treated *Cdk2^flox/flox^* mice with or without the *MvhCre^ERT2^* transgene with tamoxifen (Fig. S7 C). We observed similar increases in NRF1 target gene expression in *Cdk2^flox/flox^ MvhCre^ERT2^* but not *Cdk2^flox/flox^* mice treated with tamoxifen. This indicates that the increases in NRF1 gene expression occur in spermatocytes upon *Cdk2* deletion regardless of whether mice were treated with tamoxifen or corn oil.

### Changes in protein expression after loss of *Cdk2* in testis

Since mRNA expression is a limited readout, we decided to study the protein expression of selected NRF1/CDK2 target genes in testis and performed Western blots from extracts of inducible *Cdk2-iKO* mice. The protein expression of NRF1, histone H3, and γ-tubulin remained unchanged in all lanes (Fig. 6 B). As expected, CDK2 was not detected in *Cdk2KO* testis (Fig. 6 B, lanes 4–6; please note that in the *Cdk2-iKO* conditional knockout CDK2 is not deleted in somatic cell types [Leydig and Sertoli], which primarily express p33 CDK2). Interestingly, the protein levels of EHMT1 as well as its downstream histone modification H3K9me2 were increased after deletion of *Cdk2* compared with wild type in the inducible *Cdk2-iKO* (Fig. 6 B, lanes 4–6). These results indicate that after deletion of *Cdk2*, not only the mRNA expression of NRF1/CDK2 target genes increases but also the corresponding protein expression of at least one target (EHMT1), suggesting that this could also be true for other target genes.

### Pharmacological inhibition of EHMT1 activity decreases apoptosis in early stages *Cdk2KO* spermatocytes

With the knowledge that H3K9me2 levels are linked to the process of chromosomal synapsis (Tachibana et al., 2007), we sought to determine how inhibition of the H3K9me2 signal throughout prophase I would affect meiotic progression in spermatocytes. To study this, we used a competitive inhibitor of EHMT1/EHMT2 (UNC0642; Liu et al., 2013) to prevent H3K9me2 deposition by this methyltransferase complex. UNC0642 is effective in reducing H3K9me2 levels in vivo, and an optimal dosage scheme has been described (Kim et al., 2017). To bypass the blood–testis barrier and to increase the effectiveness of our treatments, we opted to administer UNC0642 by microinjection directly into the seminiferous tubules via the efferent duct. This method has previously been used to introduce cells (Ogawa et al., 1997), exogenous proteins (Maeda et al., 2002), and shRNA constructs (Liu et al., 2010) directly into the testis.

**Figure 7.**
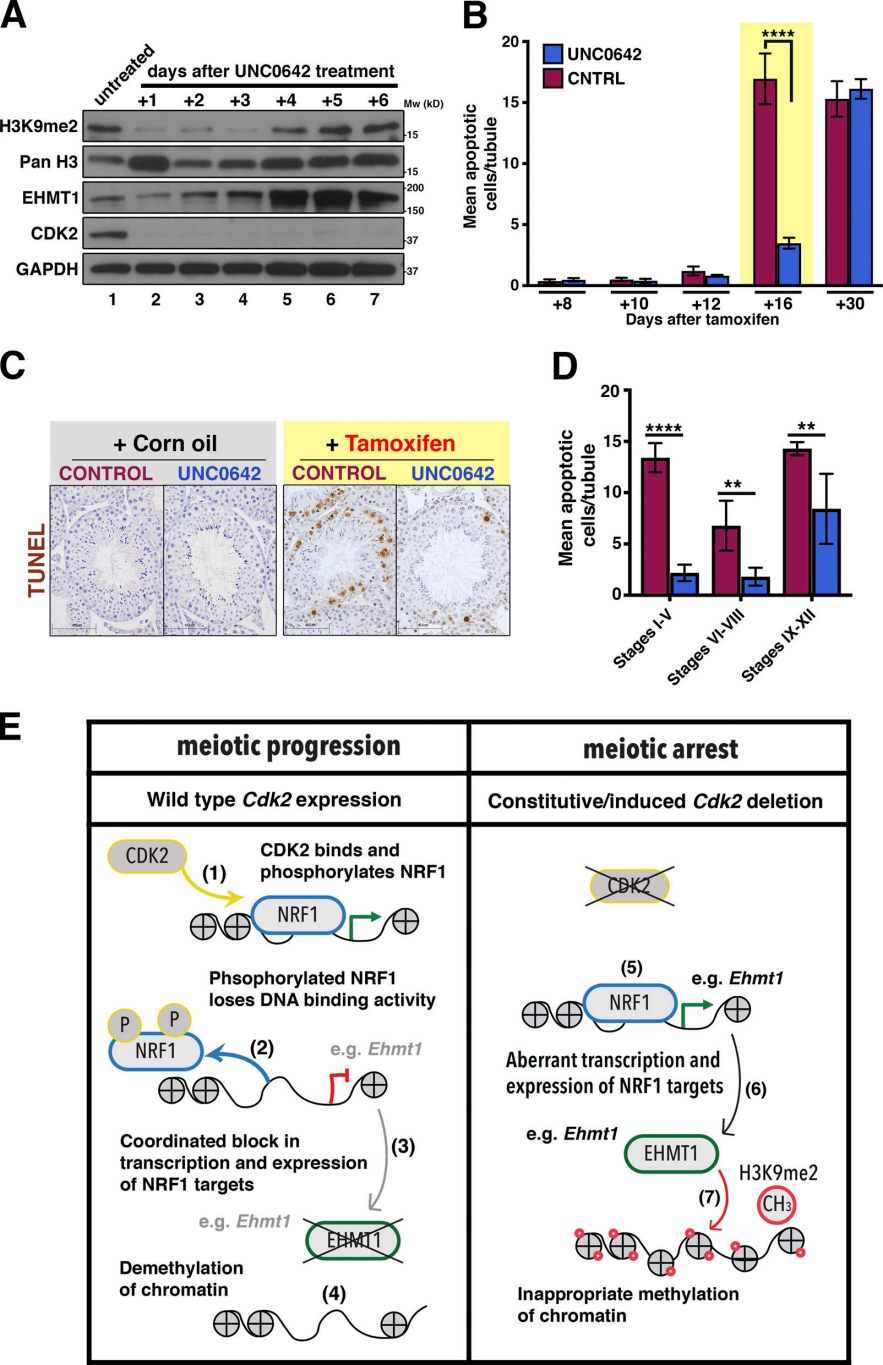
**Pharmacological inhibition of EHMT1 partially rescues the phenotype of early *Cdk2KO* spermatocytes. (A)** Time course experiment to determine the effectiveness of UNC0642 in reducing EHMT1 activity against H3K9 in *Cdk2-iKO* mice. *Cdk2-iKO* mice at P56 were injected with corn oil (lane 1) or tamoxifen (lanes 2–7), and 1 wk later were injected with UNC0642 (lanes 2–7). Whole testis lysates extracted from mice culled 1–6 d after the initial UNC0642 injection were blotted using the indicated antibodies. H3K9me2 levels were reduced by UNC0642 treatment for up to 3 d as compared with control. After this time, H3K9me2 levels were seen to rise again. *Cdk2* deletion was confirmed by the loss of CDK2 protein in all mice receiving tamoxifen (lanes 1–7). Upon *Cdk2* deletion, the expression of EHMT1 rose steadily over time in agreement that *Cdk2* is a potential negative regulator of this protein acting through NRF1. GAPDH was used as a loading control. **(B)** Apoptotic counts in induced (+ tamoxifen) *Cdk2-iKO* mice treated with UNC0642 (blue bars) or control solution (CNTRL; red bars). Apoptotic counts are measured as mean apoptotic (TUNEL-positive) cells counted per tubule. Tissues collected here follow the schedule shown in Fig. S3 A with the addition of a single microinjection of UNC0642 or vehicle control (CNTRL) solution 7 d after the first treatment of tamoxifen or corn oil. Tissue collection was then performed 8, 10, 12, 16 or 30 d after either tamoxifen or corn oil treatment. At least 50 images were counted for each biological replicate. At least three biological replicates were taken for each time point and for each condition. Values shown are presented as the mean of at least three biological replicates ± SD. **(C)** Representative TUNEL-stained images from uninduced (+ Corn oil) or induced (+ Tamoxifen) *Cdk2-iKO* mice microinjected with either UNC0642 or vehicle control solution 16 d after either corn oil or tamoxifen treatment. Hematoxylin is used as a nuclear counterstain. Scale bars, 62.7 µm. **(D)** Staging of seminiferous tubules showing apoptotic counts, in induced *Cdk2-IKO* mice treated with UNC0642 (blue bars) or without UNC0642 (red bars) 16 d after tamoxifen treatment. Tubules were classified as stages I–V, VI–VIII, or IX–XII based on the morphology of cells. Apoptotic counts are measured as mean apoptotic (TUNEL-positive) cells counted per tubule. At least 20 images were counted for each stage, for each biological replicate. Values shown are presented as mean of at least three biological replicates ± SD. For B and D, mean apoptotic cell numbers/tubule were found to have a nonnormal distribution. Significance when comparing UNC0642-treated (blue) and control-treated (red) conditions in these panels was calculated by Kruskal–Wallis one-way ANOVA (**, P ≤ 0.01; ****, P < 0.0001). **(E)** Proposed mechanism of NRF1-CDK2 interaction and how this pathway could potentially be altered in the absence of *Cdk2* to increase the expression levels of many NRF1 target genes. (1) In wild-type germ cells, chromatin-associated NRF1 is bound and phosphorylated by CDK2; (2) upon phosphorylation, NRF1 DNA-binding activity is reduced and NRF1 dissociates from chromatin. Phosphorylation here is indicated by “P” in yellow circles. (3) Upon dissociation, the transcription of NRF1 target genes such as *Msh4*, *Asz1*, and *Ehmt1* is decreased; (4) reduced *Ehmt1* expression results in a concurrent reduction in the levels of H3K9me2 on chromatin, resulting in widespread demethylation. Methylation here is indicated by “CH3” in red circles. (5) In the absence of *Cdk2* expression, NRF1 is able to bind target promoters and transcription is activated; (6) NRF1 transcriptional activity remains high, and *Ehmt1* is inappropriately transcribed; and (7) the continued expression of EHMT1 allows H3K9me2 levels to remain high. This occurs in parallel with the observation of meiotic arrest.

During validation of this treatment, we observed that in adult mice after a single dose of UNC0642, levels of H3K9me2 were reduced for at least 3 d after injection (Fig. 7 A). Induced *Cdk2-iKO* mice were treated with either a vehicle control solution or UNC0642 1 wk after tamoxifen injection, and the levels of apoptotic cells seen were subsequently quantified by TUNEL staining of testis sections. Apoptosis of germ cells served as a readout for the loss of *Cdk2* (see Fig. S3 B). As shown previously for induced *Cdk2-iKO* mice that did not receive testis microinjection (red points in Fig. S3 B), levels of apoptosis remained low until 12 d after tamoxifen treatment in both groups irrespective of treatment received (red bars in Fig. 7 B). However, increased levels of apoptosis could be observed in induced *Cdk2-iKO* mice receiving vehicle control microinjection by 16 d after tamoxifen treatment (Fig. 7 B). In contrast, induced *Cdk2-iKO* mice also receiving UNC0642 displayed significantly lower levels of apoptotic cells at the same time point (compare yellow highlighted bars in Fig. 7 B and images in Fig. 7 C). This reduction in apoptosis in UNC0642-microinjected mice, however, was not sustained, and similarly high levels of apoptosis were observed by 30 d after tamoxifen treatment in induced *Cdk2-iKO* mice irrespective of treatment, suggesting that the observed reduction was stage-specific or only transient.

To investigate in detail the reduced apoptotic levels observed in induced *Cdk2-iKO* mice receiving UNC0642 microinjection for the 16 d after tamoxifen time point, we performed approximate seminiferous staging of TUNEL-stained sections using previously described histopathological guidelines (Ahmed and de Rooij, 2009) to identify the different stages of spermatogenesis. We found that average apoptotic cell counts were decreased by 78% in stage I–V tubules and 72% in stage VI–VIII tubules, but only 37% in stage IX–XII tubules (Fig. 7 D). Our data suggest that UNC0642 treatment significantly reduced apoptosis in stage I–VIII tubules, which contain early pachytene spermatocytes, but was less effective in stage IX–XII, which contain spermatocytes in the latest stages of prophase I. This indicates to us that the EHMT1 inhibitor may delay the onset of apoptosis in early pachytene spermatocytes, but this is not sufficient to prevent their eventual apoptotic death. This likely explains why the reduction in apoptosis was not long-lived and could not be seen by 30 d after tamoxifen treatment.

Our data therefore indicate that H3K9me2 contributes to the arrest stage of *Cdk2KO* spermatocytes, but is not the only cause for the meiotic arrest, which is not surprising given the telomeric functions of CDK2 (Viera et al., 2009, 2015; Mikolcevic et al., 2016; Tu et al., 2017). Nevertheless, deregulation of EHMT1 likely contributes to the meiotic arrest in *Cdk2KO* together with the many NRF1 targets that we identified in our study. Our work suggests that CDK2 regulates meiosis not only through its telomeric functions but also by indirectly controlling the timing of H3K9me2 demethylation at the zygotene–pachytene transition through modulating the transcriptional activity of NRF1 and its many downstream targets, including *Ehmt1*.

## Discussion

In this study, we identified and characterized a novel role for CDK2 and NRF1 in the dynamic regulation of H3K9me2 during meiotic prophase I. This occurs via the modulation of *Ehmt1* transcription through the interaction of CDK2 with the NRF1 transcription factor. CDK2 was found associated with chromatin in testis and was able to bind to promoter regions also bound by the transcription factor NRF1. Our ChIP-reChIP analysis indicated that NRF1–CDK2 interactions can occur on chromatin of the identified loci. The proposed interaction between these proteins is strengthened by both the overlapping temporal and spatial expression of these two proteins during spermatogenesis and our observation that these proteins can be coimmunoprecipitated. CDK2 phosphorylates NRF1 at S136 within its DNA binding domain in vitro. Loss of *Cdk2* increased the binding of NRF1 to a number of target promoters in testis, and this was associated with broadly increased mRNA transcription from these sites. We can thus conclude that CDK2-mediated interactions contribute directly or indirectly to regulation of NRF1 DNA binding and transcriptional activity.

Canonically, NRF1 has been associated with the regulation of mitochondrial biogenesis through the transcription of essential components of the electron transport chain (Gopalakrishnan and Scarpulla, 1995). Subsequent research into tissue-specific functionality of NRF1 was limited by the fact that germline knockout leads to lethality by embryonic day 6.5 (Huo and Scarpulla, 2001). Reports of elevated expression and testis-specific expression patterns of NRF1 splice isoforms (Escrivá et al., 1999; Schaefer et al., 2000) were only further investigated upon the recent generation of a germ cell specific *Nrf1* knockout mouse model (Wang et al., 2017). In this model, deletion of *Nrf1* by the noninducible *Mvh-Cre* (*Ddx4-Cre*) resulted in spermatogonial stem cell death, preventing the onset of subsequent meiotic divisions (Wang et al., 2017). Therefore, information about specific NRF1 functions in meiotic cell types remains uncertain. Since NRF1 is expressed in spermatocytes (see Fig. S6 A), it would be of future interest to investigate how inducible loss of *Nrf1* specifically in this cell type could impact meiotic divisions. A role for NRF1 during human spermatogenesis has also been suggested since mutations in the promoter regions of a key meiotic gene, *DAZL*, lead to the loss of NRF1 binding and are associated with infertility (Teng et al., 2012).

Although we focused primarily on a single candidate target gene, *Ehmt1*, to demonstrate how NRF1-mediated transcription was mis-regulated in the absence of CDK2, it should not be inferred that NRF1 regulates meiosis only through *Ehmt1*. There were many additional NRF1/CDK2 targets that also exhibited similar changes in expression upon loss of CDK2, including *Msh4*, *Asz1*, *Syce1*, and *Tex19.1*. It is important to note that several of these targets have also been linked to infertility when their expression is altered. *Msh4*, a MutS homologue, is known to enable crossovers between homologous chromosomes during meiosis (Kneitz et al., 2000; Santucci-Darmanin et al., 2000). *Msh4* knockout mice fail to properly synapse chromosomes during pachytene stage of meiosis I (Kneitz et al., 2000) due to defects in the repair of meiotic double-strand breaks. Furthermore, a study describing the conditional loss of *Nrf1* in testis implicated the loss of *Asz1* (*Gasz1*) transcription as a causative factor in the infertility of these mice (Wang et al., 2017). Of note is that many of the genes regulated by NRF1 are expressed at high level in testis, exhibit specific localization patterns in spermatogenic cells, or have been shown to cause defects in meiotic progression when perturbed. Considering our data as a whole, it is conceivable that NRF1 modulates the expression of multiple spermatogenesis-specific genes simultaneously. Therefore, the regulation of NRF1 transcriptional activity by CDK2 is likely used to tailor the expression of specific genes in different meiotic cell types.

Histones undergo dynamic post-translational modification during several stages of germ cell development (Hammoud et al., 2014). This is especially important during the zygotene–pachytene transition during meiosis I. We aimed to uncover the upstream regulation of H3K9me2 and identified the network-like regulation of EHMT1, NRF1, and CDK2. Pharmacological inhibition of EHMT1 activity delayed apoptosis of *Cdk2KO* spermatocytes, indicating that the retention of H3K9me2 was unlikely to be the only causative factor contributing to the defects of loss of *Cdk2* in meiosis, which was not expected to start with since CDK2 is known to have additional functions during meiosis (Viera et al., 2009, 2015). We speculate that improperly paired chromosomes retaining H3K9me2 throughout the transition from zygotene to pachytene could act as a signal for the activation of a meiotic checkpoint to remove spermatocytes with asynapsed chromosomes by apoptosis. In this case, UNC0642 treatment would remove the H3K9me2 signal triggering the activation of the meiotic checkpoint. This could then effectively delay the onset of the apoptotic process in these cells. Interestingly, in some organisms such as *Caenorhabditis elegans* a phenomenon known as meiotic silencing of asynapsed or unpaired chromatin occurs when chromosomes with asynapsed regions are present. During this event, H3K9me2 decorates asynapsed chromosomes when the synapsis checkpoint becomes activated (Bean et al., 2004; Kelly and Aramayo, 2007; Lamelza and Bhalla, 2012). In mice, a similar process is known to happen with inactivation of the asynapsed stretch of the X chromosome. This is known as meiotic sex chromosome inactivation, whereby the asynapsed region of the X chromosome becomes decorated with H3K9me2 (Khalil et al., 2004).

In conclusion, we have uncovered a novel role for CDK2 and NRF1 during meiosis, which regulates the epigenetic landscape (Fig. 7 E). The expression of CDK2 modulates the transcriptional activity of NRF1 in late meiotic prophase and likely serves to repress the transcription of many NRF1 target genes including *Msh4*, *Asz1*, *Syce1*, and the chromatin regulator *Ehmt1*. The transcriptional repression of NRF1 by CDK2 results in decreases in EHMT1 and subsequently in H3K9me2. Demethylation of H3K9me2 has previously been associated with transcriptional activation. Our work provides evidence on how epigenetics, transcription, and cell cycle regulation can be coordinated during meiosis.

## Materials and methods

### Transgenic mouse lines used in this study

*Cdk2KO* (Berthet et al., 2003), *Cdk2^D145N^* (Chauhan et al., 2016), *Cdk2^T160A^* (Chauhan et al., 2016), *Cdk2^flox^* (Jayapal et al., 2015), *Speedy AKO* (Tu et al., 2017), *Sun1KO* (Lei et al., 2009), *Prdm9KO* (Hayashi et al., 2005), *Rosa26-Lox-Stop-Lox-LacZ reporter* (Soriano, 1999), and *MvhCre-ERT2* (John et al., 2008) mice have been described and were maintained on a C57BL/6 background. For all experiments using the *Cdk2* knockin, point mutants *Cdk2^D145N^* and *Cdk2^T160A^* refer to homozygous knockin animals. *Cdk2^flox/flox^ ROSA26^LSL-LacZ/LSL-LacZ^ MvhCre-ERT2* compound mutant mice (referred to in the main text as *Cdk2-iKO*) were generated by intercrossing mice harboring the relevant transgenes. Mice were housed under standard conditions, were maintained on a 12-h light/dark cycle, were fed a standard chow diet containing 6% crude fat, and were treated in compliance with the institutional guidelines for animal care and use. All experimental protocols were approved by the Animal Care and Use Committee of Biological Resource Centre at Biopolis, Agency for Science, Technology and Research, Singapore (protocol no. 171268).

### Fixation and preparation of testis tissue samples for paraffin embedding and sectioning

#### Fixation of tissues for histological staining

Excised testis tissues were pierced with a 28-gauge needle and placed into modified Davidsons solution (30% of 37% formaldehyde [11% final concentration], 15% ethanol, and 5% glacial acetic acid in H_2_O) for 16 h at 4°C. The following day, tissues were placed into histocette II cassettes (M492.2; Simport) and immersed in 70% ethanol at 4°C. Fixed tissues were stored in this manner indefinitely until further processing.

#### Paraffin embedding of testis tissues for histological sectioning

Cassettes were removed from 70% ethanol and dehydrated in successive alcohol baths for 1 h each: 85% ethanol (2×), 95% ethanol (2×), 100% ethanol (2×), and xylene (2×). After the final xylene bath, cassettes were transferred to liquid paraffin overnight at 65°C. The following day, cassettes were transferred to a second liquid paraffin bath at 65°C. From here, paraffin blocks were made for each tissue using a HistoCore Arcadia H-Heated Paraffin Embedding Station (14039357257; Leica). Paraffin blocks were stored indefinitely at RT.

#### Sectioning of testis tissues for histological staining

5-µm histological sections were cut from paraffin blocks using a microtome (RM2235; Leica). All sections made were transferred to Superfrost Plus Microscope slides (4951PLUS4; Thermo Fisher Scientific). Slides were stored indefinitely at RT until used for immunohistochemistry or immunofluorescent staining.

### Preparation of cell solution from mouse testes for meiotic chromosome spreads: Drying down method

P18 mice were euthanized, and testes were surgically excised and placed in PBS. Upon excision, the tunica albuginea was removed from each testis and discarded. From here, the seminiferous tubules of both testes were combined and lightly pulled apart using forceps in a solution of 2.2% trisodium citrate solution (75 mM). Seminiferous tubules were then moved into a hypotonic solution buffer (30 mM Tris-HCl, pH 8.8, 5 mM EDTA, 17 mM trisodium citrate dihydrate, and 50 mM sucrose) for 20 min. After 20 min, seminiferous tubules were moved into a solution of 100 mM sucrose solution, pH 8.2. Here tubules were chopped finely with a razor blade in a Petri dish until they could be easily pipetted through an uncut 200-µl pipette tip. Cells were flushed from cut tubules by repeatedly passing tubules through a 200-µl tip (30×). The now cloudy sucrose solution was then separated from the cut tubules by tilting the Petri dish to allow liquid to pool at the edges of the Petri dish. This solution was then collected and passed through a 100-µm nylon mesh cell strainer (2×). This solution was used for downstream cell spreading.

#### Preparation of fixative and coating of microscope slides

500 mg of paraformaldehyde was dissolved in 50 ml of PBS (1% final wt/vol) containing 0.33% Triton X-100 adjusted to pH 9.2. Superfrost Plus Microscope slides (4951PLUS4; Thermo Fisher Scientific) were immersed in the fixative solution to coat the surface of the slide.

#### Cell spreading procedure

A single drop of cell solution, ≈30 µl (as prepared above), was dropped from a height of 5 cm onto one of the top corners of a fixative-coated slide while the slide was held at a slight downward angle. Via gentle tilting of the slide, the cell solution was guided to coat the entire surface of the slide. When the cell solution had fully coated the slide, the slide was held flat, and the slide was humidified by blowing onto the slide 5–10× until Newton’s rings (iridescent rings) were briefly seen. The slide was then placed into a humidified box kept at 40–50°C for at least 2 h (maximally 16 h). After this incubation period, the lid to the humidified box was removed, and slides were allowed to partially dry by fanning for ≈1–2 min on a benchtop. Slides were then moved to −80°C for (indefinite) storage until staining.

### Immunofluorescent staining of testis sections/meiotic chromosome spreads

#### De-paraffinization and antigen retrieval

5 µm histological sections were de-paraffinized and rehydrated. Antigen retrieval was then performed by incubating slides in a bath of 10 mM trisodium citrate solution (71402; Sigma-Aldrich; diluted in water and adjusted to pH 6.5) at 95°C for 45 min within a water bath. Slides were then slowly cooled by removal of the 10 mM trisodium citrate solution to RT before proceeding to blocking. For meiotic chromosome spreads, the antigen retrieval step was not performed. Instead, before blocking, slides were washed once in water containing 0.1% dishwashing liquid for 5 min and subsequently three times in PBS for 5 min.

#### Blocking

Slides for all immunostaining experiments were incubated in PBS containing 10% BSA (A7906; Sigma-Aldrich), 3% milk powder (1706404; Bio-Rad), and 0.15% Triton X-100 for at least 1 h in a hydrophobic chamber at RT, under constant rocking. Blocking solution was held on slides by drawing a hydrophobic window around the area of interest using a DAKO pen (Agilent).

#### Primary antibody incubation

Primary antibodies were diluted in PBS containing 10% BSA, 3% milk powder, 0.15% Triton X-100 as stated in Table S7. Slides were incubated in primary antibody in a hydrophobic chamber for 16 h at 4°C, under constant rocking.

#### Secondary antibody incubation

Secondary antibodies were diluted in PBS containing 10% BSA, 3% milk powder, and 0.15% Triton X-100 as stated in Table S7. Slides were incubated in secondary antibody in a hydrophobic chamber for 45 min at 4°C subjected to constant rocking.

#### Washing

After blocking and primary and secondary antibody incubation steps, slides were washed three times for 5 min in PBS containing 0.5% Tween-20.

#### Nuclear counterstaining

After the wash step, following secondary antibody incubation, optional nuclear counterstaining was performed by incubating slides in Hoechst 33342 trihydrochloride trihydrate (H3570; Invitrogen) diluted 1/10,000 in PBS for 6 min. If this step was performed, slides were washed again three times for 5 min in PBS before proceeding to coverslip placement.

#### Coverslip placement and imaging

Following immunostaining, Immu-Mount aqueous immersion (9990402; Thermo Fisher Scientific) was applied to slides. Coverslips were applied and sealed with nail polish.

### Immunohistochemical detection of NRF1

5-µm histological sections were de-paraffinized and rehydrated, and antigen retrieval was performed as described for the preparation of slides for immunofluorescence imaging.

#### Blocking and antibody incubations

Slides for immunohistochemical staining were incubated in PBS containing 1% BSA for at least 1 h in a hydrophobic chamber at RT under constant rocking. For antibody incubations, NRF1 antibody was diluted in PBS containing 0.1% BSA as stated in Table S7. Slides were incubated in primary antibody in a hydrophobic chamber (1 h, under constant rocking, at RT). Secondary antibodies were then applied and detected as stated in Table S7. Here the DAB^+^ substrate was applied for ≈3 min until positive signal developed. After appropriate detection, slides were washed three times in PBS for 5 min. Stained slides were then counterstained as necessary.

#### Coverslip placement and imaging

Stained slides were dehydrated, and Eukitt (03989; Sigma-Aldrich) quick-drying mounting medium was applied to slides to affix coverslips. Slides were then stored indefinitely at RT in the dark.

### TUNEL immunostaining

TUNEL staining was performed as previously described (Wang et al., 2018). 5-µm tissue sections were de-paraffinized and rehydrated. TUNEL was performed using the ApopTag Kit (S7101; Millipore). TUNEL-stained slides were then counterstained with hematoxylin before coverslip placement and imaging.

### X-gal staining for detection of *LacZ* reporter transgene

X-gal (LacZ) staining was performed on 10-µm optimal cutting temperature compound-embedded cryosections of testis as previously described (Wong et al., 2011). Cryosections were made using a cryostat (CM3050; Leica). Prior to X-gal staining, slides containing cryosections were fixed in 4% PFA, pH 9.5, for 30 min at RT. Slides were then washed three times in detergent wash, 20 min each wash (0.1 M Na_2_HPO_4_, pH 7.3, 0.1 M NaH_2_PO_4_, pH 7.3, 2 mM MgCl_2_, 0.1% sodium deoxycholate, 0.02% NP-40, and 0.05% BSA, diluted in water). X-gal (R0404; Thermo Fisher Scientific) was reconstituted to 50 mg/ml in dimethylformamide (227056; Sigma-Aldrich). This solution was then diluted to a concentration of 0.3 mg/ml in staining solution (7.2 mM NaCl, 5 mM K_3_Fe(CN)_6_, and 5 mM K_4_Fe(CN)_6_, in detergent wash). Slides were left to stain in this solution overnight at 37°C, in the dark. After X-gal staining, slides were washed three times at RT in PBS, 20 min each, in the dark. Hematoxylin counterstaining of X-gal stained sections was performed by incubating stained sections in a 10% solution of Harris Hematoxylin (HHS32; Sigma-Aldrich) diluted in water at RT, for 10 min, in the dark. Again, slides were washed three times at RT in PBS, 20 min each, in the dark. After the final wash, slides were further incubated in 4% PFA, pH 9.5, overnight at 4°C, in the dark, to fix the stain. From here, slides were dehydrated, and Eukitt (03989; Sigma-Aldrich) quick-drying mounting medium was applied before coverslips were added. Slides were stored indefinitely at RT in the dark.

### Hematoxylin and eosin staining and hematoxylin counterstaining

Hematoxylin and eosin staining was performed as previously described (Satyanarayana et al., 2008). Incubation in undiluted Harris Hematoxylin was performed for 3 min, and incubation in eosin (HT110316; Sigma-Aldrich) was performed for 30 s dependent on the tissue section used for staining. Stained slides were washed three times for 5 min in water before proceeding to dehydration and coverslip placement.

#### For hematoxylin counterstaining of immunohistochemistry or TUNEL-stained sections

Stained slides were incubated in a 10% solution of Harris Hematoxylin diluted in water for 10 min. Counterstained slides were washed three times for 5 min in water before proceeding to dehydration and coverslip placement.

### Microscope image acquisition

#### Fluorescence imaging

All fluorescence microscope images were taken using a Zeiss AxioImager Z1 (electron beam lithography) motorized microscope. 40× magnification images were taken using an oil immersion, Plan Fluor Lens with a 1.3× numerical aperture. 100× magnification images were taken using an oil immersion Plan Apochromat lens with a 1.4 numerical aperture. All images were taken at RT using Immersol immersion oil as the imaging medium. Specific signal of primary antibodies was detected using Alexa Fluor secondary antibodies conjugated to either Alexa Fluor 488 or 500 fluorophore dyes. Images were taken using an Axio cam Hrc camera using X-cite metal halide as a fluorescence source. Images were acquired using the Zen 2.3 (blue edition) acquisition software. After imaging, further processing was performed using Adobe Photoshop CC 2018 to add pseudocolors and to overlay different channels of costained images. Where comparisions were drawn between fluorescent images, lamp intensity and exposure time was kept identical when taking images.

#### Brightfield imaging

All brightfield images were taken using an Olympus BX-51 motorized microscope. 40× images were taken using an oil immersion Plan fluor lens with a 0.75× numerical aperture. 60× images were taken using an oil immersion Plan fluor lens with a 1.25× numerical aperture. All images were taken at RT using Olympus immersion oil as the imaging medium. Images were taken using an Olympus D22 camera using a HAL 100 light source. Images were acquired using CellSens acquisition software. No further processing was performed for brightfield images.

### Protein extraction and Western blotting from cells or tissues

Dependent upon the downstream use of protein lysates, protein extraction was performed using either RIPA buffer (20 mM Tris-HCl, pH 8.0, 150 mM NaCl, 0.1% SDS, 0.5% sodium deoxycholate, 1% Triton X-100 supplemented with 10 mg/ml pepstatin, 10 mg/ml leupeptin, and 10 mg/ml chymostatin) or EBN buffer (80 mM β-glycerophosphate, pH 8.0, 10% glycerol, 15 mM MgCl_2_, 20 mM EGTA, 150 mM NaCl, 0.5% NP-40, 10 mg/ml leupeptin, 10 mg/ml chymostatin, and 10 mg/ml pepstatin, adjusted to pH 7.3 with KOH) as a lysis buffer. EBN buffer was used for any application where immunoprecipitation would be performed on the subsequent lysate. In all other cases, RIPA buffer was used as a lysis buffer. Tissues or cell pellets for protein extraction were resuspended in variable amounts of ice-cold lysis buffer and pestled 20× on ice using a Dounce tissue grinder pestle (357538; Wheaton). The resultant solution was then vortexed within an Eppendorf tube and incubated for 10 min to allow lysis to occur. Lysis was ensured by further sonication for five cycles within a Diagenode Bioruptor (high power, 30 s on, 30 s off) at 4°C. The lysed cell solution was then spun for 10 min at 18,000 × *g*, 4°C, and the supernatant was taken for protein quantification. At this step, an aliquot of concentrated lysate was stored at −80°C for future use as an input sample for any immunoprecipitation performed. Protein quantification of protein lysates was performed using the bicinchoninic acid assay (23225; Pierce), and proteins were preferentially diluted to 2 mg/ml containing a final concentration of 1× Laemmli SDS sample buffer (62.5 mM Tris, pH 6.8, 333 mM β-mercaptoethanol, 2% SDS, 10% glycerol, 100 mM DTT, and 0.01% bromophenol blue). 5–20 µg of lysate was used for Western blotting dependent upon the protein detected. Western blots were run on 8–12% polyacrylamide gels and transferred to 0.2 µm nitrocellulose membranes (1620112; Bio-Rad) via wet transfer using transfer buffer (24.7 mM Tris base, pH 8, 20% methanol, and 192 mM glycine) at 100 V for 2 h at 4°C. Nitrocellulose membranes were blocked using 4% milk powder in TBST (20 mM Tris-HCl, pH 8.0, 150 mM NaCl, and 0.1% Tween-20). Blocked membranes were incubated with specific antibody overnight as listed in Table S7. Blots were washed three times for 10 min in TBST before being incubated with HRP-conjugated secondary antibody as listed in Table S7. Blots were washed three times for 10 min in TBST before being incubated with Immobilon chemiluminescent HRP substrate (WBKLS0500; Millipore). Chemiluminescence was detected on Super RX-N X-ray films (47410; Fujifilm) and quantified by densiometric analysis using FIJI software (Schindelin et al., 2012).

### Immunoprecipitation of native proteins from whole testis lysate

1–2 mg of testis lysates extracted into EBN lysis buffer was mixed with 1–5 µg of antibody to be used for immunoprecipitation and topped up to a final volume of 1 ml using EBN buffer. This mixture was left to incubate overnight (constant rotation, 4°C). The next day, 20 µl protein A agarose beads (15918-014; Invitrogen) were added to each sample and left to incubate for a further 2 h (constant rotation, 4°C). Immunoprecipitated samples were then centrifuged (1,000 × *g* for 5 min, 4°C), and the supernatant was removed using a 30-gauge needle. protein A beads were washed three times in 1 ml of EBN buffer. Following the final wash, beads were resuspended in 55 µl of EBN buffer and 11 µl of 6× Laemmli SDS sample buffer. Samples were boiled at 95°C for 5 min. 20 µl of each sample was run on 8–12% SDS-acrylamide gels, and proteins were detected via Western blotting.

### Culture, transfection, and lysis of HEK293T cells for Western blotting and immunoprecipitation of overexpressed proteins

#### Lipofectamine 2000 transfection of HEK293T cells

In 10-cm Nunclon cell culture dishes (150464; Thermo Fisher Scientific), HEK293T cells were grown to a confluency of 70% in 15 ml of complete DMEM media (SH30243.01; GE Healthcare Life Sciences) containing 10% FBS (26140–079; Gibco) and 1% penicillin streptomycin (09367–34; Nacalai Tesque). Cell culture conditions were kept stable at 37°C, 5% CO_2_. Medium was aspirated and replaced with 15 ml of PBS. PBS was aspirated and replaced with 15 ml Opti-MEM I Reduced Serum Medium (31985062; Gibco). Plates were then left to incubate for 30 min at 37°C. For each transfection, 1–5 µg of plasmid DNA for overexpression was mixed with 100 µl of Opti-MEM in an Eppendorf tube. In a separate Eppendorf tube, 10 µl of Lipofectamine 2000 Transfection Reagent (11668019; Thermo Fisher Scientific) was mixed with 100 µl of Opti-MEM. Both tubes were then left to rest at RT for 10 min. After 10 min, the contents of both Eppendorf tubes were combined and mixed thoroughly via pipetting up and down. This combined solution was left at RT for a further 20 min. After 20 min, the combined solution was added dropwise to the cell culture plate containing Opti-MEM. The plate was swirled during addition of solution to ensure even dispersion of solution. The plate was then incubated overnight at 37°C. The next day, Opti-MEM was aspirated from the plate and replaced with 15 ml of complete DMEM. The following day (48 h after the initial transfection), cells were harvested for lysis.

#### Lysis of HEK293T cells

Complete DMEM was aspirated from cell culture plates and replaced with PBS (2×). PBS was then fully aspirated, and 100–200 µl of ice-cold EBN buffer supplemented with protease inhibitors (80 mM glycerophosphate, pH 7.3, 20 mM EGTA, 15 mM MgCl_2_, 1 mg/ml ovalbumin, 10 mg/ml pepstatin, 10 mg/ml leupeptin, 10 mg/ml chymostatin, 10 mM DTT, and 0.5% NP-40) was added to each plate. Cells were collected via detachment into the EBN buffer using a cell scraper while plates were immersed in ice. The resultant solution was then vortexed within an Eppendorf tube and incubated for 10 min to allow lysis to occur. Lysis was ensured by further sonication for five cycles within a Diagenode Bioruptor (high power, 30 s on, 30 s off) at 4°C. The lysed cell solution was then centrifuged (18,000 × *g* for 10 min, 4°C), and the supernatant was taken for protein quantification. At this step, an aliquot of concentrated lysate was stored at −80°C for future use as an input sample for any immunoprecipitation performed.

#### Immunoprecipitation procedure from HEK293T cells

Immunoprecipitation was performed by incubating HEK293T lysates with 1 µg of specific antibody as listed in Table S7 (overnight, constant rotation, 4°C). Here, the amount of protein lysate used for immunoprecipitation was altered dependent upon the downstream experiment. For immunoprecipitation of overexpressed proteins, 100–200 µg of protein lysate was typically used, and 10–20 µg of the protein lysate used for immunoprecipitation was used as input (10% of input). Regardless of the protein amount used for immunoprecipitation, 12 µl of protein A agarose (15918-014; Invitrogen) was added to the mixture for 2 h at 4°C and left to incubate under constant rotation. After 2 h, protein A beads were subsequently washed three times in 1 ml EBN buffer supplemented with protease inhibitors. Finally, protein A beads were resuspended in 60 µl Laemmli SDS sample buffer diluted to 1× in EBN buffer. The decision to boil the final solution was made dependent upon the size of the final protein to be detected. If the protein to be detected was 50 kD or lower, beads were not boiled. If the protein to be detected was >50 kD, the beads were boiled for 5 min at 95°C. For Western blotting, 10 µl of the subsequent immunoprecipitated sample was used. All antibodies used for immunoprecipitation and Western blotting are listed in Table S7.

### RNA extraction from cells and tissues

Tissues for RNA isolation were placed into 500 µl TRI Reagent (T9424; Sigma-Aldrich) in lysing matrix D tubes (69 13-500; MP Biomedicals). These tubes were then loaded into a Precellys 24 tissue homogenizer (P000669-PR240-A; Bertin Technologies), and lysis was performed for three cycles of 5 min each. The subsequent tissue lysate was mixed and centrifuged (18,000 × *g* for 10 min, at 4°C). After centrifugation, the upper layer was taken and added to 1 ml of chloroform (C2432; Sigma Aldrich). This mixture was shaken vigorously by hand and centrifuged again (18,000 × *g* for 10 min, at 4°C). The upper layer was taken and added to isopropanol (here, 500 µl of isopropanol was added per 1 ml of supernatant taken). This mixture was shaken vigorously by hand and left to precipitate at RT for 15 min. After 15 min, precipitated RNA was pelleted via centrifugation (18,000 × *g* for 10 min, at RT). The RNA pellet was subsequently resuspended and washed twice in 100% ethanol and then once in 70% ethanol. Following a final centrifugation (18,000 × *g* for 10 min, at RT), the RNA pellet was left to dry at RT for 20 min until residual ethanol surrounding the pellet had evaporated. Here the RNA pellet was resuspended in biotechnology-grade water. RNA concentration was measured using a NanoDrop 8000 spectrophotometer (ND-8000-GL; Thermo Fisher Scientific) and adjusted to 500–800 ng/µl using DNase/RNase/protease-free biotechnology-grade water (BUF-1180; First Base). This RNA solution was then snap-frozen in liquid nitrogen and stored indefinitely at −80°C until use.

### Reverse transcription of RNA and qPCR

RNA extraction, first-strand cDNA synthesis to generate cDNA, and qPCR were performed as previously described (Lim and Kaldis, 2012). A standard amount of 4 µg RNA was converted to cDNA using Maxima Reverse transcription (EP0741; Thermo Fisher Scientific). This cDNA solution was then diluted to 5 ng/µl and snap-frozen and stored indefinitely at −80°C for future use. For each qPCR reaction, 10 ng (2 µl of cDNA at 5 ng/µl) of cDNA was amplified using PowerUP SYBR green master mix (A25918; Thermo Fisher Scientific). For all qPCR experiments, cycle threshold (CT) values from experimental mice and control mice were normalized to the expression levels of the Eukaryotic elongation factor 2 housekeeping gene, and the fold change was calculated via the 2-ΔΔCT method (Livak and Schmittgen, 2001). Eukaryotic elongation factor 2 was chosen specifically due to previous publications indicating the expression stability of this gene in a wide array of murine tissues and experimental conditions (Kouadjo et al., 2007; Eissa et al., 2016). All qPCR measurements were made with either a CFX96 or CFX384 Touch Real-Time PCR Detection System (1855195/1855485; Bio-Rad). Specific primers used for all qPCR reactions shown in this study are as listed in Table S9.

### Preparation of heterogeneous spermatogenic single-cell suspensions from adult mouse testis tissue

Adult (8–12-wk-old) wild-type C57BL/6 mice were euthanized, and testes were surgically excised and placed in sterile PBS. Upon excision, the tunica albuginea was removed from each testis and discarded. From here, the seminiferous tubules of both testes were combined and lightly pulled apart using forceps.

Seminiferous tubules were then placed into a prewarmed (37°C) solution of 5 ml HyClone DMEM (SH30081.02; GE Healthcare Life Sciences) supplemented with collagenase IV (5 mg/ml; LS17104019; Gibco) and DNase I (5 mg/ml; 11284932001; Roche). This solution was then incubated for 20 min at 37°C with gentle mixing every 5 min. After 20 min, tubules were pelleted via centrifugation at 200 × *g* for 5 min, and the supernatant was removed and replaced with 5 ml of prewarmed (37°C) trypsin-EDTA (0.25%; 25200056; Gibco) supplemented with 5 mg/ml DNase I. This solution was poured into a Petri dish, and a razor blade was used to roughly mince the seminiferous tubules until they could easily be pipetted up and down within a 1-ml pipette tip. This solution was then transferred to a Falcon tube and kept in a 37°C water bath for 15 min with periodic agitation. After 15 min, 5 ml of DMEM containing 0.5% BSA was added to stop the digestion reaction. This solution was passed twice through a 100-µm nylon mesh cell strainer (22363549; Thermo Fisher Scientific). The resultant solution was spun at 200 × *g* for 5 min. Following centrifugation, the supernatant was removed. The cell pellet was resuspended in 5 ml prewarmed (37°C) DMEM and passed twice through a 100-µm nylon mesh cell strainer. The resultant cell solution was centrifuged at 200 × *g* for 5 min, and the cell pellet was resuspended in 1 ml PBS. The cell concentration in this final cell solution was then counted using a hemocytometer. If at this point the cells were to be stored for later use (either protein or nucleic acid extraction), they were pelleted again (200 × *g* for 5 min), and the pellet was snap-frozen in liquid nitrogen and stored at −80°C.

### ChIP on primary cells extracted from testis tissue

This ChIP protocol was adapted from the previously described protocol of Guccione et al. (2006). Here, heterogeneous single-cell suspensions were prepared via collagenase digestion of seminiferous tubules isolated from adult mouse testes (as stated above). Approximately 50–100 million spermatogenic cells from this heterogenous cell population were pooled and pelleted via centrifugation (200 × *g* for 5 min, RT).

#### Dual fixation with ethylene glycol bis(succinimidyl succinate) and formaldehyde

Cell pellets prepared as above were resuspended in 10 ml of prewarmed (37°C) PBS containing 1.5 mM ethylene glycol bis(succinimidyl succinate) (E3257; Sigma-Aldrich) reconstituted in DMSO (C6164; Sigma-Aldrich). Cells were left to fix at 37°C in a water bath for 10 min under constant agitation. After 10 min, 37% formaldehyde solution (252549; Sigma-Aldrich) was added to a final concentration of 1%. Cells were incubated to fix at 37°C in a water bath for a further 10 min under constant agitation. Fixation was quenched by addition of 125 mM glycine (1610718; Bio-Rad), dissolved in H_2_O. For other examples of dual fixation, refer to the studies by Tian et al. (2012) and Zeng et al. (2006). Fixed cells were pelleted via centrifugation (200 × *g* for 5 min, RT), the supernatant was removed, and cells were twice resuspended in 5 ml PBS. At this point, cells could be pelleted a final time to be snap-frozen in liquid nitrogen and stored at −80°C for use in later ChIP experiments. Alternatively, the procedure could be continued to the next step of cell lysis and sonication.

#### Lysis and sonication of fixed cells

The pellet of fixed cells was resuspended in 6 ml of ice-cold IP buffer supplemented with 10 mg/ml pepstatin, 10 mg/ml leupeptin, and 10 mg/ml chymostatin. IP buffer was preprepared from a mixture of one part SDS buffer (Tris-HCl, pH, 8.0, 100 mM NaCl, 50 mM, 5 mM EDTA, 0.02% sodium azide, and 1% SDS) and 0.5 parts Triton Dilution buffer (100 mM Tris-HCl, pH 8.0, 100 mM NaCl, 5 mM EDTA, 0.02% sodium azide, and 5% Triton X-100). The 6 ml of IP buffer cell suspension was split into six 1.5-ml Eppendorf tubes of 1 ml volume. These tubes were loaded into a Diagenode Bioruptor and sonicated at high power (30s on, 30 s off) inside a cooling water bath preset to 4°C. This was performed until chromatin was sheared to ≈300–500 bp. This occurred after roughly 16–18 cycles of sonication in the manner described above.

#### Pre-clearing and immunoprecipitation

After sonication to the desired length, the DNA concentration of the sonicated extracts was estimated from a 50-µl aliquot. DNA quantification was performed using a NanoDrop 8000 spectrophotometer (ND-8000-GL; Thermo Fisher Scientific). The concentration of the 50-µl aliquot was then used to estimate the total DNA present in the total sonicated extract. Using this estimate, the sonicated extract was then pooled into volumes containing 10 µg of genomic DNA for each immunoprecipitation to be performed and were topped up to a final volume of 1 ml using IP buffer. For each volume to be used for immunoprecipitation, 20 µl of protein A beads (15918-014; Invitrogen) was added for preclearing of the sonicated extract (1 h, constant rotation, 4°C). After 1 h, protein A beads were pelleted by centrifugation (100 × *g* for 6 min, 4°C), and the supernatant was transferred to a new tube. Immunoprecipitation was performed on the supernatant by adding 5 µg of specific antibody as detailed in Table S7, and samples were left to incubate (overnight, constant rotation, 4°C).

#### Addition and washes of protein A beads

The next day, immunoprecipitated samples were spun at 10,000 × *g* for 30 min, and the supernatant was transferred to a new tube. The supernatant was then incubated with 20 µl pre-blocked protein A beads (4 h, constant rotation, 4°C). Pre-blocked protein A beads were prepared beforehand by washing protein A beads in a solution of 1 ml lysis buffer containing 3% BSA (3×, 10 min each wash). Pre-blocked protein A beads were then stored at 4°C until use. After incubation, protein A beads were washed successively in detergent buffers as listed below and described in Guccione et al. (2006). Between each wash, beads were pelleted by centrifugation (100 × *g* for 6 min, 4°C) and the supernatant was drained using a 30-gauge needle.

#### First wash

The first wash was conducted two times in 1 ml of Mixed Micelle wash buffer (20 mM Tris-HCl, pH 8.0, 150 mM NaCl, 5 mM EDTA, 0.02% sodium azide, 0.2% SDS, 0.5% Triton X-100, 10.5% wt/vol sucrose, 10 mg/ml pepstatin, 10 mg/ml leupeptin, and 10 mg/ml chymostatin, diluted in water).

#### Second wash

The second wash was conducted two times in 1 ml of LiCl Detergent wash buffer (5 mM Tris-HCl, pH 8.0, 0.5% deoxycholic acid, 1 mM EDTA, 250 mM LiCl, 0.5% NP-40, 0.02% sodium azide, 10 mg/ml pepstatin, 10 mg/ml leupeptin, and 10 mg/ml chymostatin, diluted in water).

#### Third wash

The third wash was conducted two times in 1 ml of Buffer 500 (50 mM Hepes, pH 8.0, 0.1% deoxycholic acid, 1 mM EDTA, 1% Triton X-100, 0.02% sodium azide, 10 µg/ml pepstatin, 10 mg/ml leupeptin, and 10 mg/ml chymostatin, diluted in water).

#### Fourth wash

The fourth wash was conducted two times in 1 ml of TE buffer (10 mM Tris-HCl, pH 8.0, 1 mM EDTA, 10 µg/ml pepstatin, 10 mg/ml leupeptin, and 10 mg/ml chymostatin, diluted in water).

#### Reverse cross-linking, DNA purification, and elution

Upon the final protein A bead washing step, 260 µl of Reverse cross-linking buffer (0.1 M NaHCO_3_ and 1% SDS) containing RNase A (R4642; Sigma-Aldrich; added to a final concentration of 200 µg/ml from a stock of 10 mg/ml) was added to the beads to start the reverse cross-linking process. At this point, input samples should also be taken for reverse cross-linking. 260 µl of Reverse cross-linking buffer (0.1 M NaHCO_3_ and 1% SDS, diluted in H_2_O) containing RNase A (R4642; Sigma-Aldrich; added to a final concentration of 200 ng/ml from a stock of 10 mg/ml) was added per 50 µl of input sample. For this point onward, input samples and ChIP samples were treated in an identical manner and were incubated overnight at 65°C. The next day, Proteinase K (AM2542; Invitrogen) was added to all samples at a final concentration of 350 µg/ml (from a stock of 20 mg/ml, diluted in H_2_O) and incubated for a further 1 h at 60°C. DNA extraction was then performed directly on each sample using a QIAquick PCR Purification Kit (28104; Qiagen). At this point, eluted DNA was either used to check the lengths of sonicated DNA or stored for downstream applications.

#### For downstream ChIP-qPCR/ChIPseq

ChIP DNA was eluted from the QIAquick PCR Purification Kit in 40 µl H_2_O. This was snap-frozen in liquid nitrogen and stored at −80°C until further use.

### ChIP-qPCR assay

Genomic ChIP DNA and input DNA were extracted as described for ChIP. Equal volumes of purified genomic ChIP DNA and (1%) input DNA were amplified using PowerUP SYBR green master mix (A25918; Thermo Fisher Scientific) as described for qPCR. Enrichment of binding was calculated via comparison of the amplification seen for 1% of input genomic DNA used for the original ChIP assay (percentage of recovered input). Percentage of recovered input was determined using the following calculation: [100 × 2^ (adjusted input − Ct (IP)]. A full list of oligonucleotide sequences used for ChIP-qPCR assays is shown in Table S10.

### ChIP-reChIP assay

ChIP-reChIP assays were performed using the method of Furlan-Magaril et al. (2009). Here, ChIP-reChIP was performed using antibodies as listed in Table S7. For this protocol isolation, fixation and quenching of fixative were performed in the same manner as that described for ChIP. Following these steps, fixed cells were centrifuged (200 × *g* for 5 min, 4°C) to pellet cells, and this cell pellet was washed twice in ice-cold PBS. Following the second wash, cells were pelleted and resuspended in 1 ml of lysis buffer and incubated on ice for 10 min. Lysis and sonication were performed as described for ChIP. Following sonication, an aliquot of input DNA was taken and stored at −20°C until use. Sheared chromatin for immunoprecipitation was then diluted in dilution buffer (20 mM Tris-HCl, pH 8.1, 1% Triton X-100, 2 mM EDTA, and 150 mM NaCl).

#### Immunoprecipitation with antibody 1

For the first immunoprecipitation step, aliquots were made for (lane 1) NRF1, (lane 2) IgG, (lane 3) CDK2, and (lane 4) nonspecific IgG immunoprecipitation (Fig. 4 E and Fig. S2, A–C). 5 µg of each antibody was added to each aliquot and left to incubate (overnight, constant rotation, 4°C). Pre-blocked protein A beads were then prepared as described for ChIP, 20 µl of protein A beads was added to each immunoprecipitation condition, and samples were left to mix (4 h, constant rotation, 4°C).

#### Protein A bead wash I

Protein A beads were pelleted by centrifugation (800 × *g* for 5 min, 4°C) and were washed sequentially in 1 ml of wash buffer I (20 mM Tris-HCl, pH 8.1, 0.1% SDS, 1% Triton X-100, 2 mM EDTA, 150 mM NaCl, 10 mg/ml pepstatin, 10 mg/ml leupeptin, and 10 mg/ml chymostatin, diluted in water), 1 ml of wash buffer II (20 mM Tris-HCl, pH 8.1, 0.1% SDS, 1% Triton X-100, 2 mM EDTA, 500 mM NaCl, 10 mg/ml pepstatin, 10 mg/ml leupeptin, and 10 mg/ml chymostatin, diluted in water), and 1 ml of wash buffer III (10 mM Tris-HCl, pH 8.1, 0.25 M LiCl, 1% NP-40, 1% deoxycholate, 1 mM EDTA, 10 mg/ml pepstatin, 10 mg/ml leupeptin, and 10 mg/ml chymostatin, diluted in water). Beads were then washed twice in 1 ml TE buffer (10 mM Tris-HCl, pH 8, 1 mM EDTA, 10 mg/ml pepstatin, 10 mg/ml leupeptin, and 10 mg/ml chymostatin, diluted in water) and resuspended in TE buffer containing 10 mM DTT. Samples were left to elute in this buffer at 37°C for 30 min. Following elution, samples were centrifuged (800 × *g* for 2 min, 4°C) and the supernatant was diluted 20× using dilution buffer.

#### Immunoprecipitation with antibody 2

For the second immunoprecipitation step, CDK2 antibodies were added (lanes 1 and 2) and NRF1 antibodies were added (lanes 3 and 4) to the immunoprecipitation. As a control, additional aliquots for singular ChIP were made and NRF1 (lane 5), CDK2 (lane 6), nonspecific IgG (lane 7) antibodies were added and incubated overnight at 4°C with constant rotation (Fig. 4 E and Fig. S2, A–C).

#### Protein A bead wash II

The steps for the second protein A bead wash are identical to those described for the first protein A bead wash.

#### Reverse cross-linking, DNA purification, and elution

Reverse cross-linking, DNA purification, and elution were identical to those described for the ChIP protocol.

#### PCR amplification of ChIP-reChIP DNA

PCR amplification of ChIP-reChIP samples was performed in the same manner as described for ChIP-qPCR.

### ChIPseq and bioinformatic analysis

For ChIPseq analysis, DNA libraries were prepared using the TruSeq ChIP Sample Prep kit (P-202-1012; Illumina), following the manufacturer’s instructions, and sequenced on the Illumina HiSeq 2000 and NextSeq 500 platforms at Genome Institute Singapore. The sequenced reads were mapped to the mm9 build of the mouse genome using the aligner Bowtie2 version 2.2.6 (Langmead et al., 2009) with default parameters. Only reads that mapped uniquely to the genome with at most one mismatch were kept. Duplicate reads were filtered out by MACS (2.0.9; Zhang et al., 2008) to limit PCR-induced biases, and the q value was set to 0.05 for MACS peak calling. To further select high-confidence peaks, the minimum fold change was set to three. For additional analysis, GREAT INPUT (McLean et al., 2010) and MEME SUITE software (Bailey and Elkan, 1994; Gupta et al., 2007) were used to identify overrepresented sequences in ChIPseq datasets and generate motifs used for figures. Both NRF1 and CDK2 ChIPseq datasets were repeated at least three times with similar results. Only one dataset from each was shown and analyzed within this study.

### Plasmid construction and site mutagenesis

The full-length human *Nrf1* isoform 1 (NM_001040110.1) coding sequence was transferred from a Human ORFeome V8.1 Library (OHS7588; Dharmacon) via a LR clonase reaction using Gateway LR Clonase Enzyme mix (11791019; Thermo Fisher Scientific) into a pENTR/D-TOPO entry vector (k240020; Thermo Fisher Scientific). From there, the *Nrf1* sequence was sub-cloned into either the PGEX6P1 vector for expression of GST-tagged fusion proteins in *Escherichia coli* or a lentiviral packaging vector (p-Bobi) containing a MYC tag. For the cloning of GST-NRF1 peptide coding sequences and their mutants, oligonucleotides as listed in Table S11 were annealed and digested before ligation into the PGEX6p1 vector. For the creation of full-length NRF1 mutants, serine-to-alanine substitution mutations were introduced into plasmid DNA by PCR-based site-directed mutagenesis using the QuikChange II Site-Directed Mutagenesis Kit (200523; Agilent). All primers used for sub-cloning, site mutagenesis, and sequencing of *Nrf1* and mutant *Nrf1* sequences are listed in Table S11.

### Recombinant GST-fusion protein expression and purification

#### Preparation of bacterial cultures for expression of GST-fusion proteins

Dh5α *E. coli* cells (18265017; Invitrogen) transfected with plasmids for GST-fusion protein expression were grown to an OD of 0.5–0.7 (600 nm absorbance) in a total volume of 1 liter lysogeny broth supplemented with 10 µg/ml ampicillin in a shaking bacterial incubator (37°C, 800 rpm). Upon reaching the correct OD, bacterial flasks were transferred to ice and cooled to 20°C. Once cooled, bacterial flasks were transferred to a shaking bacterial incubator (20°C, 800 rpm) for 20 min.

#### IPTG induction of *E. coli*

IPTG (15529019; Thermo Fisher Scientific) was added from a stock solution of 105 mM (25 mg/ml) to a final concentration of 1 mM, and bacterial flasks were incubated for 16 h at 20°C on a shaker.

#### Bacterial lysis

Bacterial cells were pelleted, and the bacterial pellet was washed with 0.9% NaCl solution, 10 mg/ml pepstatin, 10 mg/ml leupeptin, and 10 mg/ml chymostatin diluted in water. Bacterial cells were pelleted again, and the bacterial pellet was resuspended in 25 ml of lysis buffer (10% glycerol, 0.5 M NaCl, 1 mM DTT, 10 mg/ml pepstatin, 10 mg/ml leupeptin, and 10 mg/ml chymostatin diluted in PBS). Bacterial lysis was then performed using a French press at 18,000 lb/in^2^ pressure and clarified by centrifugation at 25,000 rpm for 45 min at 4°C. The supernatant was transferred to 50-ml Falcon tubes, and 10% NP-40 was added to a final concentration of 1%. This was then incubated for a further 10 min on ice.

#### Purification of GST-fusion proteins

Dependent upon the size of the initial bacterial culture, 500 µl to 3 ml of glutathione beads (16101; Pierce) was added to the bacterial lysate to bind GST-fusion proteins and left to mix at 4°C for 2 h within the 50-ml Falcon tube. Next, bacterial lysate containing the glutathione beads was added to a chromatography column (732-1010; Bio-Rad) to capture the beads, and unbound lysate was allowed to drain from the beads. Captured glutathione beads were then washed by the addition of sequential wash buffers. Five batches of each of 100 ml of wash buffer I (0.5 M NaCl, 0.5% NP-40, 1 mM DTT, 10 mg/ml pepstatin, 10 mg/ml leupeptin, and 10 mg/ml chymostatin diluted in PBS) and 100 ml of wash buffer II (0.5 M NaCl, 1 mM DTT, 10 mg/ml pepstatin, 10 mg/ml leupeptin, and 10 mg/ml chymostatin, diluted in PBS) were applied. GST-fusion proteins were then eluted from glutathione beads by the addition of elution buffer (100 mM L-glutathione reduced [G4251; Sigma-Aldrich], 133 mM NaOH, 666 mM NaCl, 133 mM Tris base, and 3 mM DTT). To ensure full elution, elution buffer was usually passed over the beads at least twice.

#### Concentration and storage of GST-fusion proteins

The eluate from the glutathione beads was then added directly to Ultra centrifugal filter units 10K (901024; Bio-Rad), 30K (910096; Bio-Rad), or 100K (903024; Bio-Rad) with the cut-off dependent on the size of the GST-fusion protein to be purified. Amicon ultra concentrators were spun at 5,000 × *g* until the level of liquid inside the concentrator had fallen to <500 µl. 1 ml of dilution buffer (50 mM Hepes, pH 8.0, containing 10% glycerol, 150 mM NaCl, 1 mM DTT, 10 mg/ml pepstatin, 10 mg/ml leupeptin, and 10 mg/ml chymostatin) was then added, and the concentrator was spun again until the level of liquid inside the concentrator had fallen to <500 µl. This step was repeated a further three times. The length of the last spin was adjusted to leave variable volumes of sample within the concentrator. This is experiment dependent and was adjusted based on the concentration of GST-fusion protein yielded in each case. Purified GST-fusion proteins were snap-frozen in liquid nitrogen and stored at −80°C until use. A full list of the plasmids used to express GST–NRF1 peptide fusion proteins or full-length GST–NRF1 fusion proteins and the amino acid sequence of GST-tagged NRF1 peptides are presented in Table S12.

### Kinase assays

Kinase assays were performed as previously described (Diril et al., 2016). 1 µg of recombinant GST–NRF1 fusion protein was incubated with 100 ng of active CDK2/cyclin A2 complexes (see below), 30 µM ATP (10127523001; Roche), and 5 µCi of [γ-^32^P] ATP (NEG502A; PerkinElmer) in EBN without NP-40, as described above for immunoprecipitation, for 30 min at RT.

#### Preparation of active CDK2/cyclinA2 complexes

Active CDK2/cyclin A2 complexes (as referenced in the main text) refers to a mixture of baculoviral-expressed CDK2 and bacterially expressed GST-cyclin A2 and GST-Cdk-ctivating kinase. Unlike monomeric CDK2, this complex has functional kinase activity against known CDK2 substrates. Active CDK2/cyclin A2 complexes were made in house. 0.1 µg baculoviral-expressed CDK2 and 2.73 ng purified GST-Cdk-activating kinase were incubated in EBN without NP-40 containing 1 mM ATP for 4.5 h at RT. 4.4 µg of GST-purified GST-cyclin A2 was then added to the mixture and left for an additional 30 min at RT. This mixture was snap-frozen in liquid nitrogen and stored at −80°C until use.

#### Quenching and detection of kinase assay reactions

Kinase assays were quenched via the addition of 6× Laemmli SDS sample buffer to a final concentration of 1×, followed by boiling at 95°C for 5 min. Proteins were separated by SDS-PAGE on 8% polyacrylamide gels, and gels were dried using a Speedgel gel dryer (SG210D; Thermo Fisher Scientific) at 80°C for 2 h. Levels of incorporated radioactivity within dried gels were then quantified using a nonconfocal variable mode laser scanner/PhosphoImager (Typhoon FLA-7000; Fujifilm) and the Multi Gauge software (Ver3.X).

### EMSA

EMSAs were performed using DIG-labeled double-stranded DNA probes prepared as described (Lim et al., 2017). For binding assays in which GST-NRF1 was prephosphorylated by CDK2, proteins were prepared as described in the Kinase assays section, before incubation with double-stranded DNA probes. For each reaction, 10 ng of DIG-labeled *Ehmt1* promoter was incubated with 1 µg of Poly[d(I-C)] for 5 min at RT in binding buffer (5 mM Hepes, pH 8.0, 20 nM DTT, and 30 mM NaCl). 0.5–2 µg of recombinant GST-NRF1 was then added and incubated at RT for 5 min to allow specific binding to occur. Probe-protein mixtures were run on a native 6% nondenaturing Tris-borate-EDTA (TBE)–PAGE in 0.5× TBE buffer, pH 8.3 (B52; Thermo Fisher Scientific), which resulted in a final concentration of 45 mM Tris base, 45 mM borate, and 1 mM EDTA. Gels were run for 3 h at 100 V, 4°C. DIG-labeled probes were then transferred to a nylon membrane hybond-N^+^ (45-000-850; GE/Amersham) using a semidry transfer apparatus run at 100 V for 15 min. Probes were cross-linked to the nylon membrane by exposure to UV light for 30 s using a UV transilluminator 2000 (1708110edu; Bio-Rad). Before detection, nylon membranes were blocked using DIG blocking reagent (11585762001; Roche) and washed twice for 5 min in wash buffer containing 0.1 M maleic acid, pH 7.5, and 0.15 M NaCl diluted in PBS. DIG-labeled probes were detected using anti-DIG-alkaline phosphatase antibody provided in the DIG Oligonucleotide 3′-End Labeling Kit, second generation (03353575910; Roche), at a dilution of 1/10,000 in blocking buffer. Nylon membranes were washed again twice for 5 min in 0.1 M maleic acid, pH 7.5, and 0.15 M NaCl. The CDP-Star Chemiluminescent Substrate (C0712; Sigma-Aldrich) was then added for 1 min to allow detection via chemiluminescence. Chemiluminescence was detected on Super RX-N X-ray films (47410; Fujifilm) and quantified by densiometric analysis using FIJI software (Schindelin et al., 2012) in an identical manner as would be used for Western blotting. A full list of oligonucleotide sequences used for EMSA assays is shown in Table S8.

### Induction of *Cdk2* deletion in *Cdk2-iKO* mice

For induction of Cre-ERT2, 2 mg tamoxifen (T5648; Sigma-Aldrich) dissolved in corn oil (C8267; Sigma-Aldrich) was administered intraperitoneally as five 150-µl injections. These injections were given over 6 d with one rest day on the fourth day. For control treatments, corn oil without tamoxifen was given using the same schedule. Tamoxifen or corn oil treatments were always started when mice were 56 d old.

### Testis microinjection protocol

Seminiferous tubule microinjection was performed as previously described (Ogawa et al., 1997; Kanatsu-Shinohara et al., 2004). A small incision was made in the abdomen of the mouse, and testes were exposed by pulling the abdominal fat pad. Testes were injected with using a micro-capillary pipette injector (made in house). Surgical incisions were closed using 12-mm Silkam sutures (762075; Braun). 5 µg UNC0642 or control solution (SML1037; Sigma-Aldrich) was administered into both testes of injected mice. Injection volumes were adjusted to suit the capacity of tissues to retain the solution at each time point, with no more than 1 µl/mg of testis weight of either control solution or UNC0642 injected per testis. To make working dilutions of UNC0642, DMSO was first used to dissolve the initial lyophilized drug, and then this solution was further diluted in PBS to the desired concentration. Here, control solution refers to the diluent used to dissolve the UNC0642 (PBS containing trace amounts of DMSO).

### Spermatocyte isolation by STA-PUT BSA gradient system

Pachytene stage spermatocytes were isolated using the STA-PUT methodology as previously detailed (Bryant et al., 2013; Liu et al., 2015b). Validation of this method by our lab was performed via Western blotting of spermatocyte-specific markers. The purity of fractions used for experiments was confirmed both via immunostaining of isolated cells for SYCP3 and via their morphology under light microscope.

### Statistical analysis

All experiments were repeated at least three times. All statistical tests were performed using GraphPad Prism version 6 (GraphPad Software), and differences were considered significant when P < 0.05. Specific statistical tests for individual experiments are mentioned in their respective figure legends.

### Online supplemental material

Fig. S1 shows the genomic analysis of CDK2- and NRF1-binding sites. Fig. S2 shows ChIP-reChIP analysis of CDK2 and NRF1. Fig. S3 shows the validation of germ cell–specific *Cdk2* knockout in -treated *Cdk2-iKO* mice. Fig. S4 shows the validation of NRF1 as a candidate CDK2 substrate by mass spectrometry analysis and in vitro kinase assays. Fig. S5 shows the comparison of CDK2 and NRF1 ChIPseq binding sites proximal to the *Ehmt1* promoter. Fig. S6 shows the NRF1 expression during spermatogenesis. Fig. S7 shows that NRF1 target gene expression is increased upon induced *Cdk2* deletion in spermatocytes. Table S1 lists the CDK2-bound regions of chromatin in whole testis lysate as determined by ChIPseq. Table S2 lists the overlap of CDK2-bound regions of chromatin in whole testis lysate as determined by ChIPseq with a previously published E2F1 ChIPseq dataset. Table S3 lists the NRF1-bound regions of chromatin in whole testis lysate as determined by ChIPseq. Table S4 lists the overlap of NRF1-bound regions of chromatin in whole testis lysate as determined by ChIPseq with a previously published NRF1 ChIPseq dataset. Table S5 lists the overlap of CDK2- and NRF1-bound regions of chromatin in whole testis lysate as determined by ChIPseq. Table S6 lists the gene ontology of genomic loci associated with NRF1-binding in whole testis lysate. Table S7 lists the antibodies used. Table S8 lists the oligonucleotides used to create EMSA probes. Table S9 lists the oligonucleotides used for RT-qPCR against cDNA. Table S10 lists the oligonucleotides used for ChIP RT-qPCR against genomic DNA targets. Table S11 lists the oligonucleotides used for cloning. Table S12 lists the plasmids and bacterial clones used in this study.

## Supplementary Material

Supplemental Materials (PDF)

Tables S1-S12 (ZIP)
