# Supplementary material for: CDK2 regulates the NRF1/Ehmt1 axis during meiotic prophase I
Source: J Cell Biol. 2019 Jul 26;218(9):2896–918. doi: 10.1083/jcb.201903125 (PMC6719441; doi:10.1083/jcb.201903125)
Supplement: Supplemental Materials (PDF) [file JCB_201903125_sm.pdf]

Supplemental material

Palmer et al., https://doi.org/10.1083/jcb.201903125

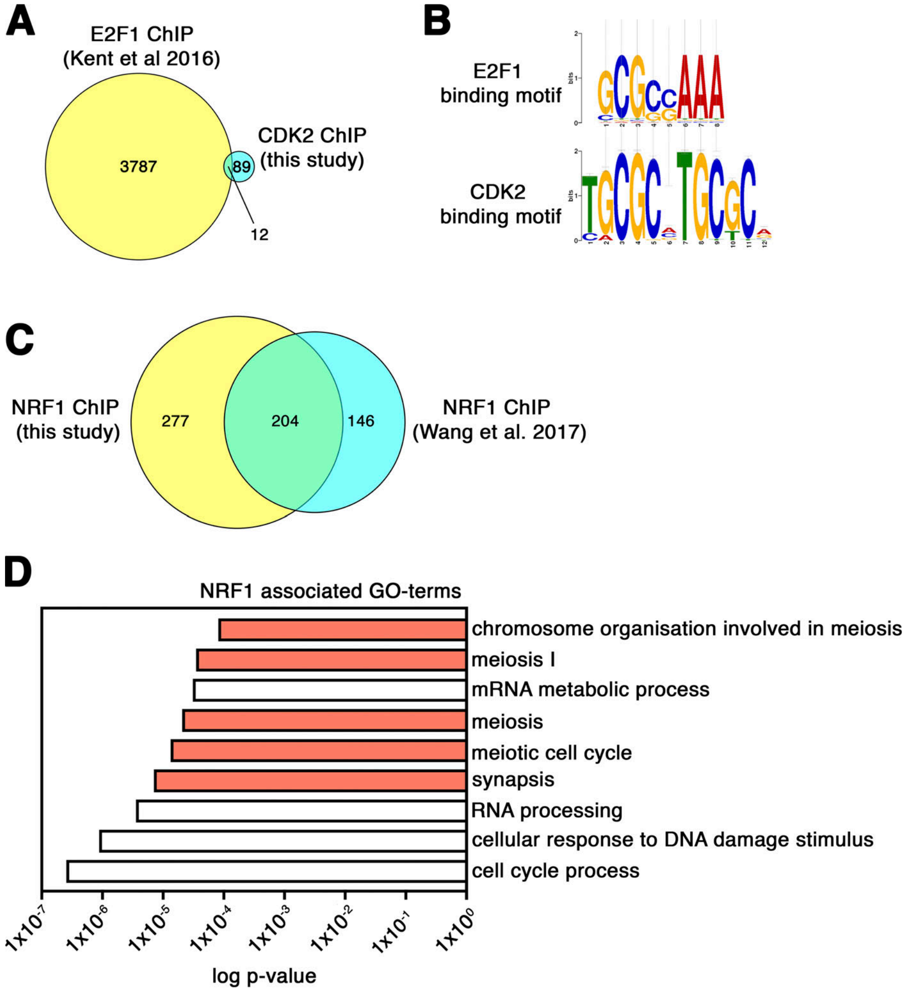

Figure S1. **Genomic analysis of CDK2 and NRF1 binding sites; related to Fig. 4. (A)** Venn diagram displaying the overlap of bound genomic regions between a previously published E2F1 ChIPseq performed on mouse embryonic fibroblasts stably overexpressing E2F1 (deposited in GEO under accession no. GSM1833208) and the CDK2 ChIPseq from our study. **(B)** Best-fit motif comparison between the experimentally determined E2F1 transcription factor binding motif deposited in the JASPAR open-access database of transcription factor binding motifs (JASPAR ID: MA0024.1; Khan et al., 2018) and the CDK2 binding motif identified in our study (shown in reverse complement). Analysis using TOMTOM software of MEME Suite (http://meme-suite.org/) indicates that these motifs are not significantly similar (P = 0.515). **(C)** Venn diagram displaying the overlap of bound genomic regions between a previously published NRF1 ChIPseq performed on mouse whole testis lysate (data extracted from Table S2 of Wang et al., 2017; blue) and the NRF1 ChIPseq from our study (yellow). Similarity of identified NRF1 binding motif in NRF1 ChIPseq (this study): 4.8473e-63; and identified NRF1 binding motif in NRF1 ChIPseq (Wang et al., 2017): 1.5911e-75, as determined by GREAT INPUT (McLean et al., 2010). **(D)** The most significant pathways (Gene ontology terms [GO-terms]) associated with NRF1-bound genomic regions are displayed with associated adjusted P-values as determined by the GREAT bioinformatics tool. Gene ontology terms associated with meiosis are shaded in red.

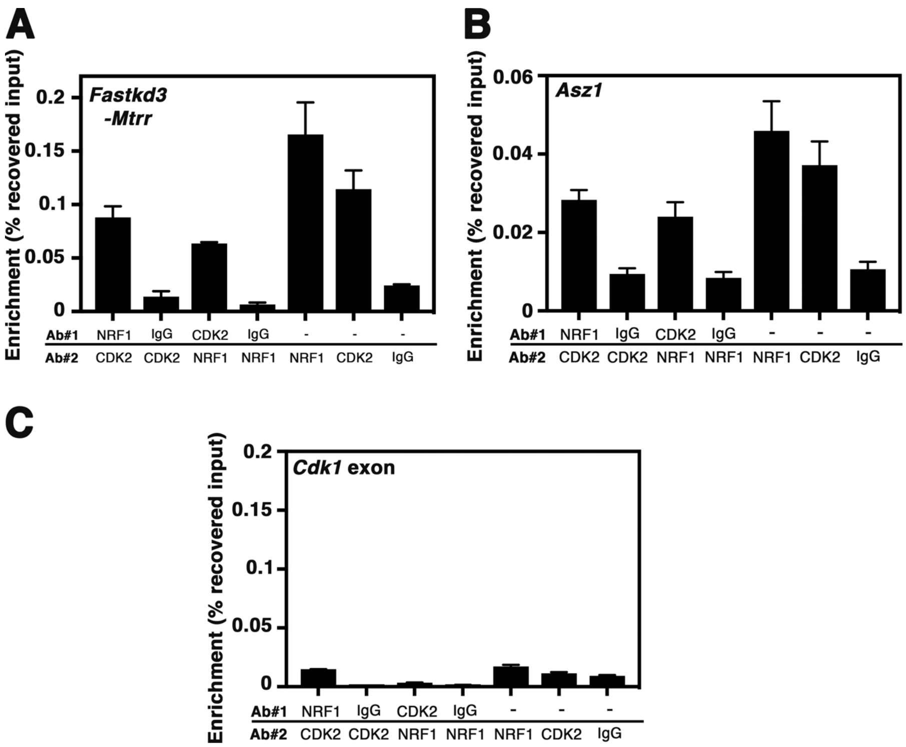

Figure S2. **ChIP-reChIP analysis of CDK2 and NRF1; related to Fig. 4.** Two ChIP-reChIPs were performed using either CDK2 or NRF1 antibodies followed by the reciprocal antibody as indicated. Control immunoprecipitations were also performed using nonspecific rabbit IgG antibodies followed by CDK2 or NRF1 antibodies. Immunoprecipitated genomic DNA was used for qPCR directed against the region of the *Fastkd3-Mtrr* promoter (A), the *Asz1* promoter (B), or an exonic region of the *Cdk1* gene (C) as negative control. Standard ChIP was also performed by using either NRF1 or CDK2 antibodies to perform a single immunoprecipitation as a positive control for binding. Non-specific rabbit IgG ChIP is also shown as a negative control. Error bars for ChIP-reChIP data are representative of the SD of at least three technical replicates of the same experiment. All ChIP-qPCR and ChIP-reChIP experiments were repeated three times using pooled spermatogenic cells from distinct groups of animals (biological replicates) with similar results.

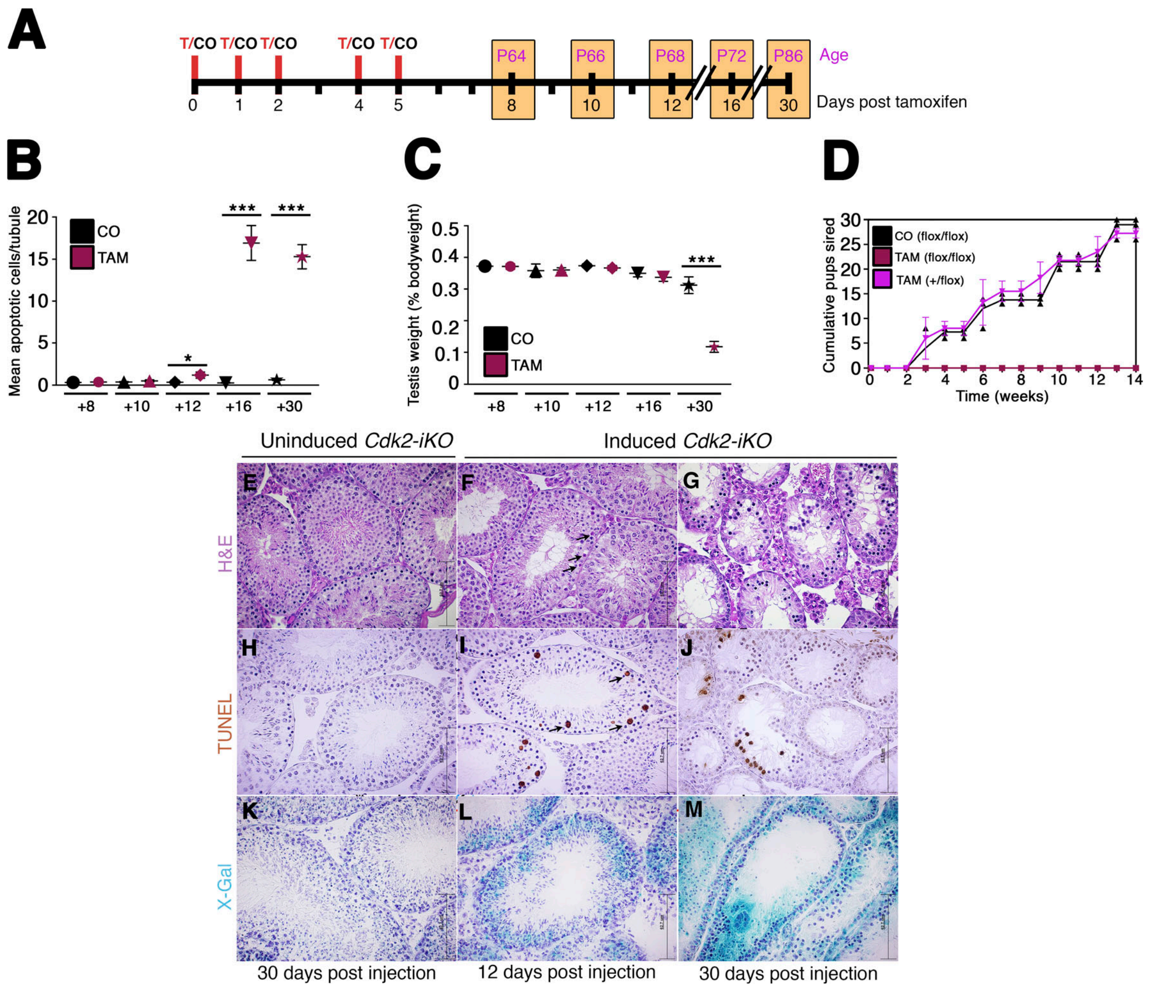

Figure S3. **Validation of germ cell specific *Cdk2* knockout in tamoxifen treated *Cdk2-iKO* mice; related to Figs. 4, 6, and 7.** Our aim was to investigate the impact of CDK2 on the functionality of NRF1 in vivo using a mouse model system with a deletion of *Cdk2*. Unfortunately, direct comparisons drawn between wild-type and germline *Cdk2KO* testis are limited by the differential cellularity caused by germ cell death in the adult *Cdk2KO* mouse (Berthet et al., 2003; Ortega et al., 2003). We therefore developed a tamoxifen-inducible, testis-specific *Cdk2* knockout mouse model (*Cdk2$^{flox/flox}$ ROSA26$^{LSL\text{-}LacZ/LSL\text{-}LacZ}$ MvhCre-ERT2*). In this model, Cre-ERT2 is expressed specifically in germ cells from the *Vasa/Mvh* promoter and localizes to the nucleus in a tamoxifen-dependent manner (John et al., 2008) to delete CDK2. For simplicity, these mice are hereafter referred to as *Cdk2-iKO*. Using this model, experimental mice were analyzed shortly after tamoxifen administration, when we observed complete deletion of *Cdk2* but before the majority of germ cells undergo apoptotic cell death. Timeline of tamoxifen/corn oil treatments given to *Cdk2-iKO* mice (A). T indicates tamoxifen treatment and was initiated when mice were 56 d old (day 0). Testis were collected 8, 10, 12, 16 or 30 d later for histological evaluation when mice were 64, 66, 68, 72 or 86 d old, respectively. Additional cohorts of *Cdk2-iKO* mice were also treated with corn oil (CO) in the same manner as controls (noninduced *Cdk2-iKO*). Testes were collected at identical time points from these mice for comparison. Apoptotic counts measured as mean apoptotic (TUNEL-positive) cells counted per tubule (B). At least 50 images were counted for each biological replicate. At least three biological replicates were taken for each time point and for each condition (tamoxifen-treated [red symbols] or corn oil–treated [black symbols]). Bodyweight to testis weight ratios, measured as the mean testis weight (g) as a percentage of the bodyweight (g) of each mouse, are displayed in C. All values measured here are of mice isolated for the experiment as shown in B (tamoxifen-treated [red symbols] or corn oil–treated [black symbols]). Fertility testing of induced and noninduced *Cdk2-iKO* mice (D). Here flox/flox denotes homozygosity for LoxP-flanked *Cdk2* alleles. Induced *Cdk2-iKO* male mice (red) or uninduced *Cdk2-iKO* male mice (black) were mated with a single C57BL/6 female mouse for a 14-wk period. Mating was started only 30 d after treatment with either corn oil or tamoxifen. Cumulative number of mice sired by mice in each condition is shown over a 14-wk period. *Cdk2$^{+/flox}$* MvhCreERT2 (+flox) mice treated with tamoxifen are shown as an additional purple line as a control for the potential effects of tamoxifen on fertility. Importantly, we noted that tamoxifen injection did not affect the fertility of heterozygous *Cdk2$^{+/flox}$* mice, indicating that this treatment was not responsible for the apoptotic cell death seen in the homozygous *Cdk2$^{flox/flox}$* mice (D). For B and C, mean apoptotic cell numbers/tubule were found to have a nonnormal distribution. Significance when comparing tamoxifen-treated (red) and corn oil–treated (black) was calculated by Kruskal–Wallis one-way ANOVA (*, $P < 0.05$; ***, $P < 0.001$). Fertility data shown in D was assumed to follow a normal distribution. Significance when comparing tamoxifen-treated +/flox (pink) and corn oil–treated flox/flox (black) groups was calculated by one-way ANOVA (not significant at all time points measured). Histological staining from selected *Cdk2-iKO* mice (E–M). Hematoxylin and eosin (H&E), TUNEL, and X-Gal staining are used to demonstrate the relative cellularity, apoptosis levels, and Cre-ERT2 induction in induced *Cdk2-iKO* at 12 (F, I, and L) or 30 d (G, J, and M) after tamoxifen treatment (middle and right panels). Images from an uninduced *Cdk2-iKO* testis 30 d after corn oil injection are also shown as control images (E, H, and K). Error bars for B–D are representative of the SD of data taken from at least three biological replicates. All images are taken at 40× magnification. Scale bars, 62.7 µm.

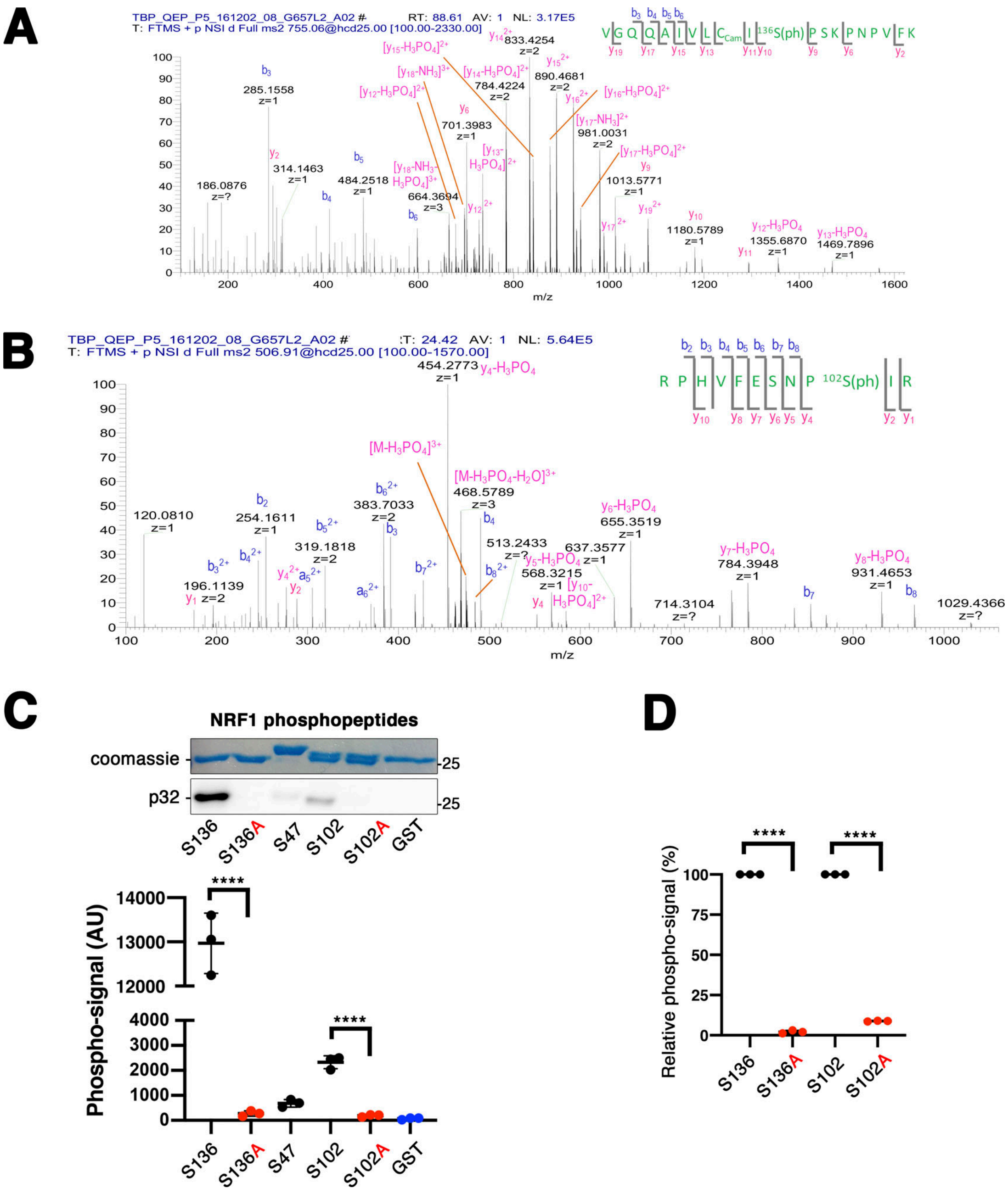

Figure S4. **Validation of NRF1 as a candidate CDK2 substrate by mass spectrometry analysis and in vitro kinase assays; related to Fig. 5.** Full-length NRF1-GST was expressed in bacteria, purified, and phosphorylated in vitro with CDK2/cyclin A2. Kinase reactions were quenched, separated by SDS-PAGE, trypsin-digested, and analyzed by liquid chromatography-tandem mass spectrometry. Phosphopeptides corresponding to S136 (A) and S102 (B) of NRF1 are shown. AU, arbitrary unit. **(C)** To validate the phosphorylation sites, we cloned short peptide sequences containing either the putative S102 or S136 phosphosites upstream of a GST tag in tandem with peptide sequences in which these serines had been mutated to a nonphosphorylatable alanine residue (S102A, S136A). We also included a peptide sequence corresponding to S47 of NRF1 due to an observation in a previous study that this site can be phosphorylated by cyclin D–containing CDK complexes (Wang et al., 2006). Kinase assay using CDK2/cyclin A2, NRF1–peptide–GST fusion proteins, and radiolabeled ATP are shown in C. A GST construct without insert (GST) was included as a negative control. The quantification of phospho-signal is displayed below as determined by PhosphoImager using the Multi Gauge software (Ver3.X). Phospho-signal data were assumed to be normally distributed, although this was not formally tested. Our data demonstrate that CDK2 phosphorylated NRF1 at both S136 and S102 yet did not have activity toward S47. **(D)** Quantification of relative phospho signal between serine-alanine mutants and wild-type phosphopeptides corresponding to S136 (left) and S102 (right) of NRF1. For B and C, significance was determined by one-way ANOVA (****, P ≤ 0.0001).

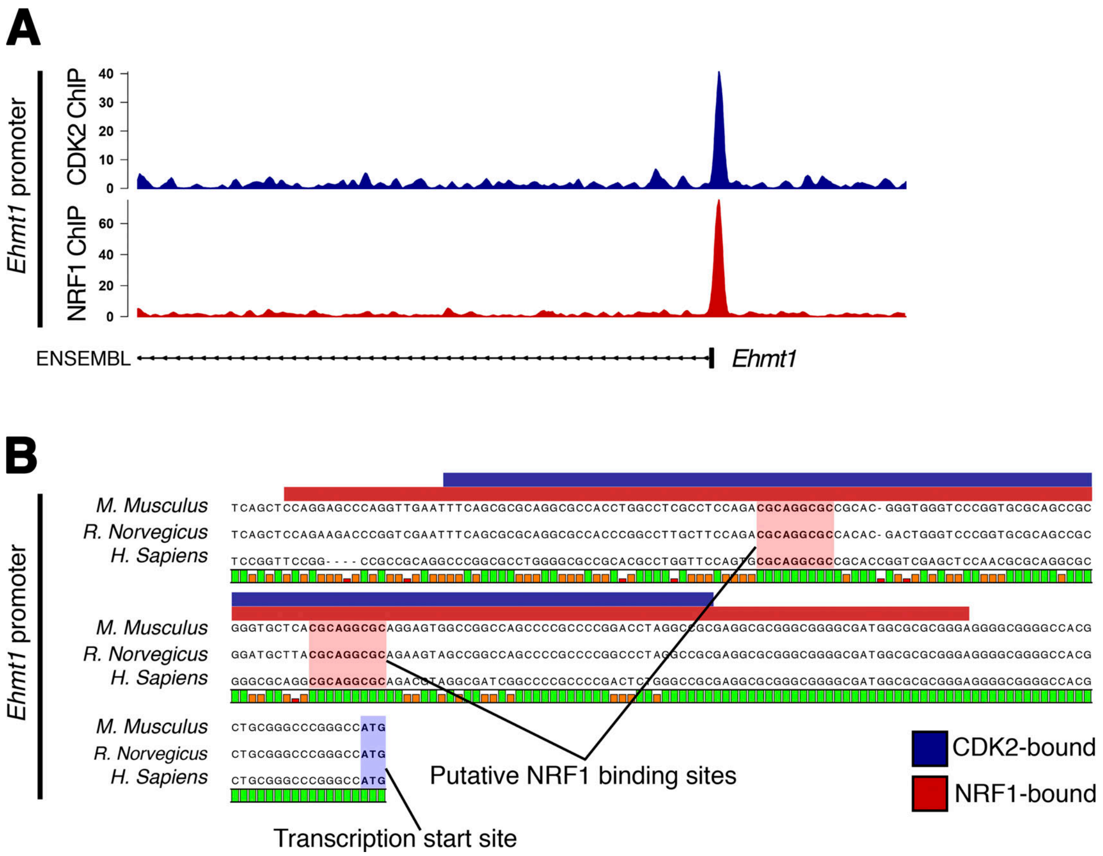

Figure S5. **Comparison of CDK2 and NRF1 ChIPseq binding sites proximal to the *Ehmt1* promoter; related to Figs. 4 and 5. (A)** Genomic tracks showing ChIPseq data for CDK2 (upper) and NRF1 (lower) across a 1-kb region in chromatin extracted from wild-type whole testis lysate. The ENSEMBL gene track is shown below the profiles. **(B)** Conservation of the DNA sequence of the *Ehmt1* promoter between the mouse, rat, and human genomes. CDK2-bound and NRF1-bound regions as determined via ChIPseq are illustrated by the blue and red bars, respectively. Two putative NRF1-binding sites are indicated by pink shading and show 100% conservation. The transcription start site is indicated by blue shading.

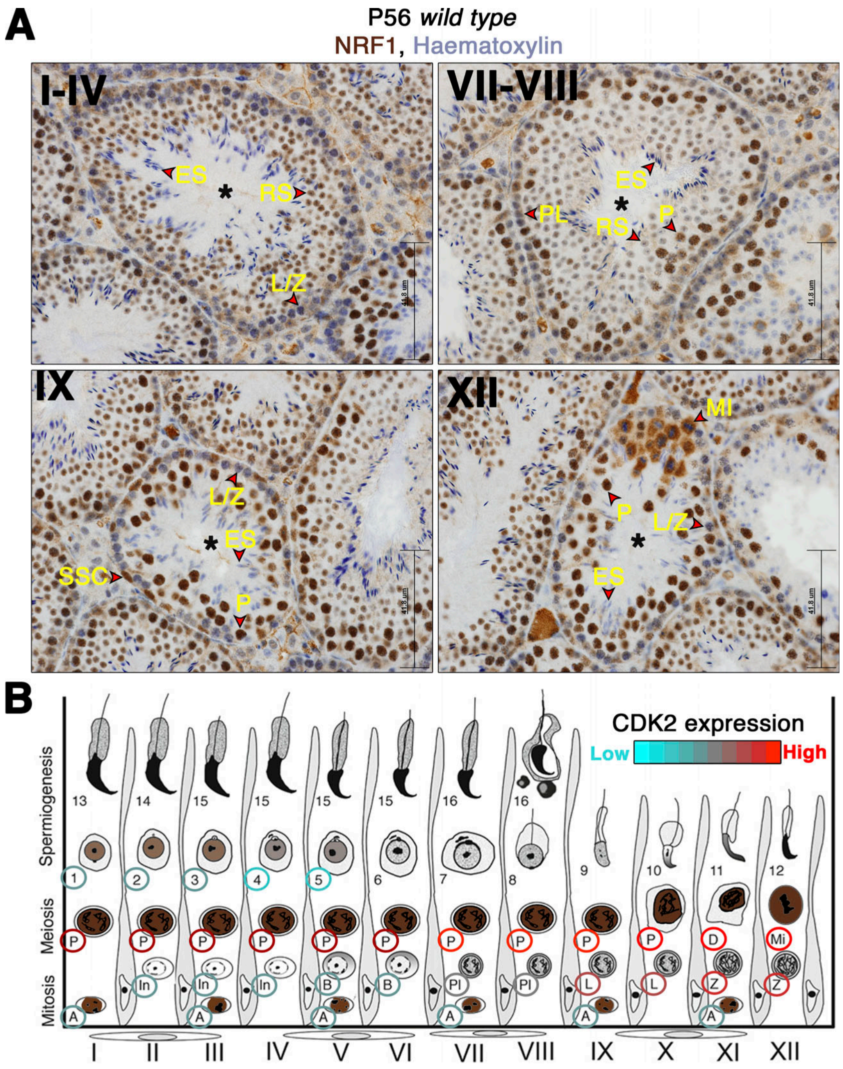

Figure S6. **NRF1 expression during spermatogenesis. (A)** Immunohistochemical staining of NRF1 in P56 wild-type testis sections. Cells positive for NRF1 signal show brown staining. Hematoxylin counterstain is used to visualize nuclei. The estimated seminiferous stage of each displayed tubules is shown by roman numerals in the top left-hand corner. Asterisks are used to denote the tubule staged in each picture. Staging was performed in reference to the graphic shown in B. Cell types identified by morphology are labeled in yellow and marked by red arrows. SSC, spermatogonial stem cell; PL, preleptotene spermatocyte; L/Z, leptotene/zygotene spermatocyte; P, pachytene spermatocyte; MI, metaphase I spermatocyte; RS, round spermatid; ES, elongating spermatid. **(B)** Graphic adapted from Cheng (2014), demonstrating the seminiferous stages of spermatogenesis as observed in an adult mouse. Using the pictures detailed in A, cell types positive for NRF1 signal have been colored in brown. Colored rings around cell type labels indicate the approximate intensity of CDK2 expression in each cell type. Cell types are as labeled for A. Additional labels refer to the following cells: A, ln, and B, spermatogonial stem cells; D, diplotene spermatocyte; Mi, metaphase I spermatocyte. Positivity for any seminiferous stage for NRF1 staining was only indicated after evaluating similar NRF1 staining for at least 30 images of an approximated stage. All images are taken at 40× magnification. Scale bars, 62.7 µm.

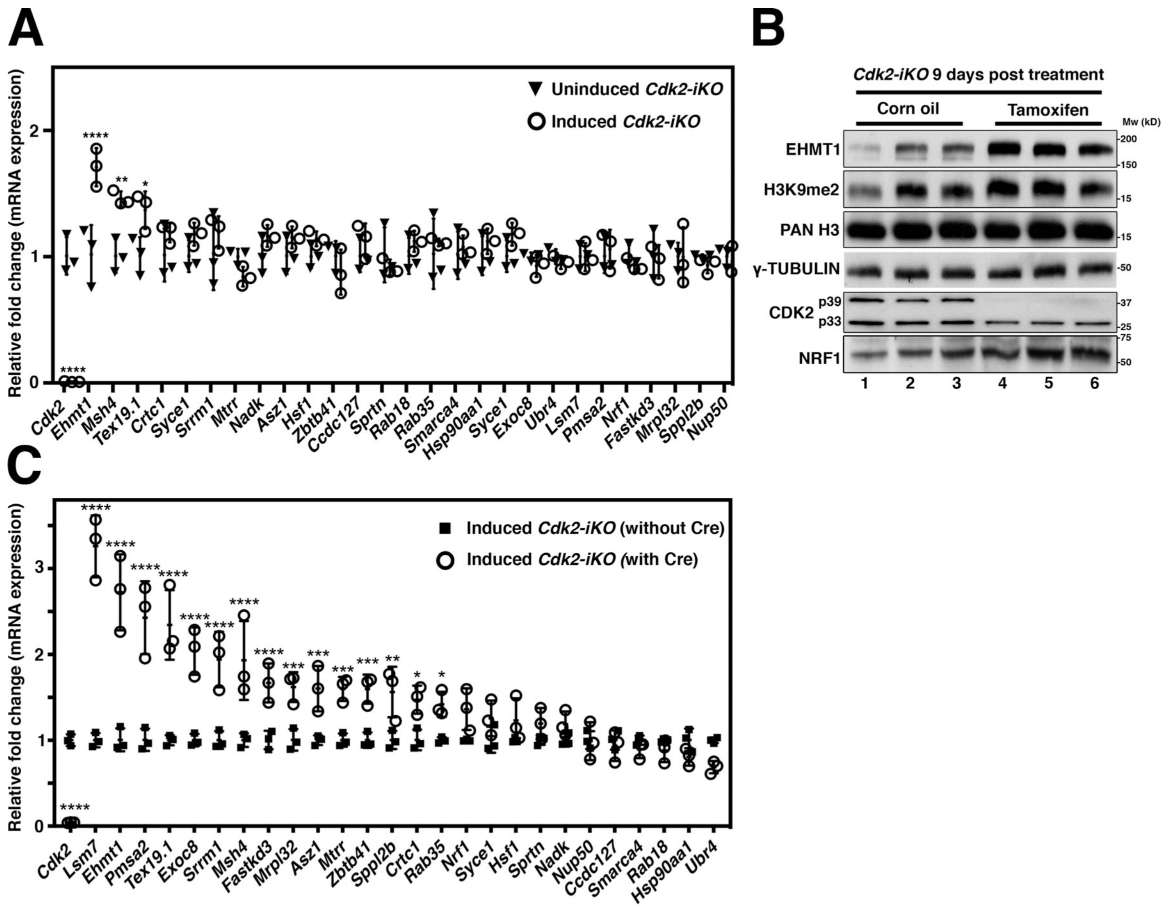

Figure S7. **NRF1 target gene expression is increased upon induced *Cdk2* deletion in spermatocytes; related to Fig. 6. (A)** qPCR analysis of spermatocyte cDNA isolated from corn oil–treated (black triangles) and tamoxifen-treated (white circles) *Cdk2-iKO* mice—referred to as noninduced and induced *Cdk2-iKO*, respectively. Expression is displayed as fold change normalized to the expression of the eEF2 housekeeping gene. STA-PUT spermatocyte isolation (as stated in Materials and methods) for this experiment was performed at P65, 9 d after corn oil/tamoxifen treatment (started at P56). 25 NRF1 target genes in addition to *Cdk2* and *Nrf1* were tested for changes in expression. 3/25 NRF1 target genes showed significant increases in expression (****, $P \leq 0.0001$; **, $P < 0.005$; *, $P < 0.05$). Error bars are representative of the SD of normalized fold change values from at least three biological replicates as compared to noninduced *Cdk2-iKO* controls. Gene expression data were assumed to be normally distributed, although this was not formally tested. Significance was determined by one-way ANOVA. Three biological replicates were tested for each group for each gene. **(B)** Western blotting of NRF1, EHMT1, and H3K9me2 in adult, corn oil–treated *Cdk2-iKO* (lanes 1–3) or tamoxifen-treated *Cdk2-iKO* (lanes 4–6) whole testis lysates extracted from three biological replicates. Testis isolation for this experiment was performed at postnatal day 65, 9 d after corn oil/tamoxifen treatment (started at P56). γ-Tubulin and PAN H3 are shown as loading controls. In tamoxifen-treated *Cdk2-iKO* animals, p33 CDK2 is detectable as this isoform is expressed in somatic cells, which do not express *MvhCre-ERT2*. The p39 isoform of CDK2 is expressed in meiotic cells which express *MvhCre-ERT2* and is therefore deleted as expected. EHMT1 protein levels are increased upon induced deletion of CDK2, which was associated with increased levels of H3K9me2. **(C)** qPCR analysis of spermatocyte cDNA isolated from tamoxifen-treated, *Cdk2*$^{flox/flox}$ mice with (white circles) and without (black squares) the *MvhCre-ERT2* transgene—referred to as induced *Cdk2-iKO* with or without Cre, respectively. Expression is displayed as fold change normalized to the expression of the eEF2 housekeeping gene. STA-PUT spermatocyte isolation (as stated in Materials and methods) for this experiment was performed at P68, 12 d after corn oil/tamoxifen treatment (started at P56). 25 NRF1 target genes in addition to *Cdk2* and *Nrf1* were tested for changes in expression. 15/25 NRF1 target genes showed significant increases in expression: (****, $P \leq 0.0001$; ***, $P < 0.001$; **, $P < 0.005$; *, $P < 0.05$). Error bars are representative of the SD of normalized fold change values from at least three biological replicates as compared induced *Cdk2-iKO* (without cre) controls. Gene expression data were assumed to be normally distributed, although this was not formally tested. Significance was determined by one-way ANOVA. Three biological replicates were tested for each group for each gene.

Provided online are 12 supplemental Excel tables. Table S1 lists of Cdk2-bound regions of chromatin in whole testis lysate as determined by ChIPseq. Table S2 shows overlap of Cdk2-bound regions of chromatin in whole testis lysate as determined by ChIPseq with a previously published E2F1 ChIPseq dataset showing E2F1-bound regions of chromatin in mouse embryonic fibroblasts. Table S3 lists the NRF1-bound regions of chromatin in whole testis lysate as determined by ChIPseq. Table S4 lists the overlap of NRF1-bound regions of chromatin in whole testis lysate as determined by ChIPseq with a previously published NRF1 ChIPseq dataset. Table S5 lists the overlap of CDK2- and NRF1-bound regions of chromatin in whole testis lysate as determined by ChIPseq. Table S6 lists the gene ontology of genomic loci associated with NRF1-binding in whole testis lysate. Table S7 lists the antibodies used. Table S8 lists the oligonucleotides used to create EMSA probes. Table S9 lists the oligonucleotides used for RT-qPCR against cDNA. Table S10 lists the oligonucleotides used for ChIP RT-qPCR against genomic DNA targets. Table S11 lists the oligonucleotides used for cloning. Table S12 lists the plasmids and bacterial clones used in this study.

References

Berthet, C., E. Aleem, V. Coppola, L. Tessarollo, and P. Kaldis. 2003. Cdk2 knockout mice are viable. *Curr. Biol.* 13:1775–1785. https://doi.org/10.1016/j.cub.2003.09.024

Cheng, C.Y. 2014. *Molecular mechanisms in spermatogenesis.* Springer, New York, NY.

John, G.B., T.D. Gallardo, L.J. Shirley, and D.H. Castrillon. 2008. Foxo3 is a PI3K-dependent molecular switch controlling the initiation of oocyte growth. *Dev. Biol.* 321:197–204. https://doi.org/10.1016/j.ydbio.2008.06.017

Khan, A., O. Fornes, A. Stigliani, M. Gheorghe, J.A. Castro-Mondragon, R. van der Lee, A. Bessy, J. Chèneby, S.R. Kulkarni, G. Tan, et al. 2018. JASPAR 2018: update of the open-access database of transcription factor binding profiles and its web framework. *Nucleic Acids Res.* 46:D260–D266. https://doi.org/10.1093/nar/gkx1126

McLean, C.Y., D. Bristor, M. Hiller, S.L. Clarke, B.T. Schaar, C.B. Lowe, A.M. Wenger, and G. Bejerano. 2010. GREAT improves functional interpretation of cis-regulatory regions. *Nat. Biotechnol.* 28:495–501. https://doi.org/10.1038/nbt.1630

Ortega, S., I. Prieto, J. Odajima, A. Martín, P. Dubus, R. Sotillo, J.L. Barbero, M. Malumbres, and M. Barbacid. 2003. Cyclin-dependent kinase 2 is essential for meiosis but not for mitotic cell division in mice. *Nat. Genet.* 35:25–31. https://doi.org/10.1038/ng1232

Wang, C., Z. Li, Y. Lu, R. Du, S. Katiyar, J. Yang, M. Fu, J.E. Leader, A. Quong, P.M. Novikoff, and R.G. Pestell. 2006. Cyclin D1 repression of nuclear respiratory factor 1 integrates nuclear DNA synthesis and mitochondrial function. *Proc. Natl. Acad. Sci. USA.* 103:11567–11572. https://doi.org/10.1073/pnas.0603363103

Wang, J., C. Tang, Q. Wang, J. Su, T. Ni, W. Yang, Y. Wang, W. Chen, X. Liu, S. Wang, et al. 2017. NRF1 coordinates with DNA methylation to regulate spermatogenesis. *FASEB J.* 31:4959–4970. https://doi.org/10.1096/fj.201700093R
